# An experimental and computational study of CO_2_ adsorption in the sodalite-type M-BTT (M = Cr, Mn, Fe, Cu) metal–organic frameworks featuring open metal sites†
†Electronic supplementary information (ESI) available: Adsorption isotherms and its crystallographic information, additional figures, Fourier difference maps, and results from DFT simulations. The refined structures by powder diffraction: CCDC 1582001–1582011. For ESI and crystallographic data in CIF or other electronic format see DOI: 10.1039/c8sc00971f


**DOI:** 10.1039/c8sc00971f

**Published:** 2018-04-23

**Authors:** Mehrdad Asgari, Sudi Jawahery, Eric D. Bloch, Matthew R. Hudson, Roxana Flacau, Bess Vlaisavljevich, Jeffrey R. Long, Craig M. Brown, Wendy L. Queen

**Affiliations:** a Institute of Chemical Sciences and Engineering , École Polytechnique Fédérale de Lausanne (EPFL) , CH-1051 Sion , Switzerland . Email: wendy.queen@epfl.ch ; Tel: +41 216958243; b Department of Chemical and Biomolecular Engineering , University of California , Berkeley , California 94720 , USA; c Department of Chemistry , University of California , Berkeley , California 94720 , USA; d Department of Chemistry and Biochemistry , University of Delaware , Newark , Delaware 19716 , USA; e National Institute of Standards and Technology , Center for Neutron Research , Gaithersburg , Maryland 20899 , USA; f Canadian Neutron Beam Centre , National Research Council , Chalk River Laboratories , Chalk River, Ontario K0J 1P0 , Canada; g Department of Chemistry , University of South Dakota , Vermillion , South Dakota 57069 , USA; h Division of Materials Sciences , Lawrence Berkeley National Laboratory , Berkeley , California 94720 , USA; i Department of Chemical Engineering , University of Delaware , Newark , Delaware 19716 , USA

## Abstract

We present a comprehensive investigation of the CO_2_ adsorption properties of an isostructural series of metal–organic frameworks, M-BTT (M = Cr, Mn, Fe, Cu; BTT^3–^ = 1,3,5-benzenetristetrazolate), which exhibit a high density of open metal sites capable of polarizing and binding guest molecules. Coupling gas adsorption measurements with *in situ* neutron and X-ray diffraction experiments provides molecular-level insight into the adsorption process and enables rationalization of the observed adsorption isotherms. In particular, structural data confirms that the high initial isosteric heats of CO_2_ adsorption for the series are directly correlated with the presence of open metal sites and further reveals the positions and orientations of as many as three additional adsorption sites. Density functional theory calculations that include van der Waals dispersion corrections quantitatively support the observed structural features associated with the primary and secondary CO_2_ binding sites, including CO_2_ positions and orientations, as well as the experimentally determined isosteric heats of CO_2_ adsorption.

## Abstract 2

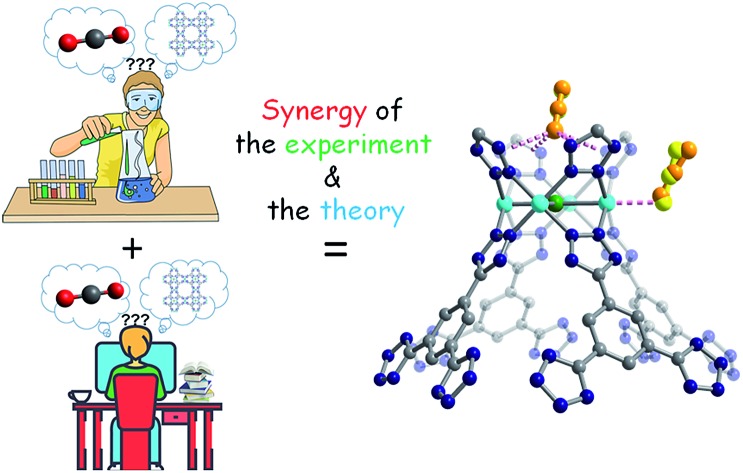
The work provides molecular level insight into the CO_2_ adsorption properties of an isostructural series of MOFs, known as M-BTT.

## Introduction

Rising levels of atmospheric CO_2_ are heavily implicated in global warming and predominantly result from the combustion of carbon-based fuels, which has steadily increased since the industrial revolution.^1^ Carbon dioxide levels recently surpassed an alarming level of 400 ppm,^2^ and given the expectation that the transition to clean, renewable energies will continue to progress slowly,^3^ the quest for new carbon capture technologies has moved to the forefront of scientific research.^4^ At present, widespread implementation of the most prominent capture technology, namely liquid amine-based scrubbers, is limited by the high regeneration energies associated with using these materials in a post-combustion CO_2_ capture process. It has been alternatively proposed that solid adsorbents, which exhibit both lower heat capacities and heats of adsorption, could reduce this parasitic energy cost considerably by requiring far less heat for regeneration.^5^,^6^

Metal–organic frameworks are a broad class of solid adsorbents consisting of metal ions linked by organic ligands to form porous, crystalline arrays, and are being intensively investigated for various energy-relevant applications, including gas storage, gas separation, and catalysis. These materials possess significant advantages over other solid adsorbents, including unprecedented internal surface areas, impressive structural diversity, and facile chemical tunability. Given the numerous permutations of potential linker and metal combinations, a vast number of frameworks are accessible in principle; however, to identify and better tune the properties of any framework for a target application, such as CO_2_ capture, it is essential to first understand structure-derived function. One powerful approach is the study of isostructural families of frameworks differing only in the identity of the metal ion.^7^–^9^ For example, we have previously shown that a combined experimental and theoretical approach can afford important insights into the factors influencing CO_2_ adsorption in the M_2_(dobdc) (M = Mg, Mn, Fe, Co, Ni, Cu, Zn; dobdc^4–^ = 2,5-dioxido-1,4 benzenedicarboxylate) series of frameworks.^10^ However, a lingering practical challenge to the conduction of such structure–property relationship studies is the need for reliable experimental adsorption and *in situ* diffraction data for a group of isostructural frameworks, which is often not available for many frameworks of interest.

**Fig. 1:**
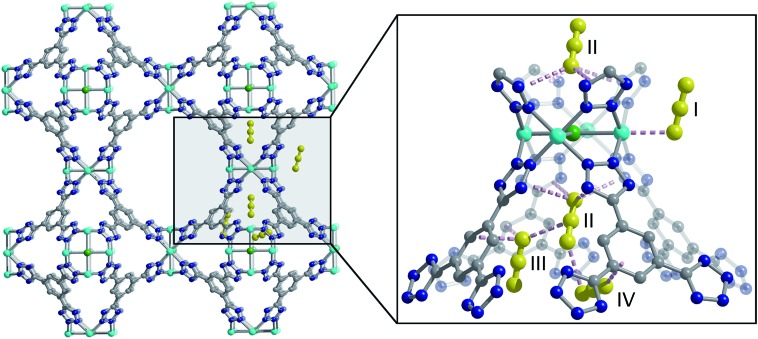
Structure of a portion of the Cu-BTT framework, as determined from Rietveld analysis of high-resolution powder neutron diffraction data (left), and an expanded view of the four different CO_2_ adsorption sites within the framework (right). Cyan, grey, blue, and green spheres represent Cu, C, N, and Cl atoms, respectively, and yellow ball-and-stick models represent CO_2_; H atoms and charge balancing cations are omitted for clarity. At site I, the Cu–O distance is 2.60(3) Å while other nearest-neighbour CO_2_-framework and CO_2_–CO_2_ interactions are dominated by van der Waals forces and range approximately from 2.95 to 3.44 Å.

One such family is the sodalite-type series M-BTT (M = Cr, Mn, Fe, Co, Ni, Cu, Cd; BTT^3–^ = 1,3,5-benzenetristetrazolate),^11^–^15^ with a general formula of [(M_4_Cl)_3_(BTT)_8_]^3–^. The framework crystallizes in the cubic *Pm*3*m* space group (no. 221) and features truncated, octahedral cages built up of six [M_4_Cl]^7+^ units and eight [BTT]^3–^ ligands that are further interlinked to form an anionic, porous, three-dimensional network (Fig. 1). This material is somewhat unique among metal–organic frameworks in that it readily undergoes chemical substitution with a number of first row transition metals and has a high density of open metal sites that are accessible after heating the as-synthesized material under dynamic vacuum to remove coordinated solvent. These properties are exhibited by only a few other known framework families, namely M_2_(dobdc),^16^–^25^ M_2_(dobpdc) (M = Mg, Mn, Fe, Co, Ni, Zn; dobpdc^4–^ = 4,4′-dioxidobiphenyl-3,3′-dicarboxylate),^26^ M_2_Cl_2_(bbta) (H_2_bbta = 1*H*,5*H*-benzo(1,2-*d*:4,5-*d*′) bistriazole) and M_2_Cl_2_(btdd) (H_2_btdd = bis(1*H*-1,2,3-triazolo[4,5-*b*],[4′,5′-*i*])dibenzo[1,4]dioxin),^27^ M_2_(*m*-dobdc) (M = Mg, Mn, Fe, Co, Ni; *m*-dobdc^4–^ = 4,6-dioxido-1,3-benzenedicarboxylate),^28^ and M_3_(btc)_2_ (M = Cr, Cu, Zn, Mo, Ru; btc^3–^ = 1,3,5-benzene tricarboxylate).^29^–^32^

The open metal sites of the M-BTT family have been shown previously to increase the surface packing density of hydrocarbon adsorbates,^33^ to exhibit strong and selective binding of framework guests,^13^,^15^,^34^,^35^ and also to provide a pathway to achieve charge transfer between frameworks and guest species,^36^ a desirable property for using metal–organic frameworks for the conversion of small molecules into value-added chemicals.^37^ Most relevant to this study, the open metal sites can improve CO_2_ uptake at low-pressures, which is a property of interest for flue gas separations in post-combustion capture technologies.^6^,^38^ The M-BTT family thus stands as an important system for experimental examination of the role of metal identity on CO_2_ adsorption and subsequent identification and validation of computational tools useful in varying chemical environments.

Herein, we present a comprehensive experimental and computational investigation of CO_2_ adsorption in M-BTT (M = Cr, Mn, Fe, Cu) analogs, using *in situ* powder neutron diffraction and periodic density functional theory (DFT) calculations in tandem. Powder neutron diffraction reveals for the first time a molecular level view of CO_2_ adsorption in M-BTT and facilitates rationalization of experimental CO_2_ adsorption isotherms. The experimentally obtained binding distances and enthalpies are further used to benchmark and evaluate the results of DFT calculations on M-BTT, as reported in this work and a previous study.^35^ Ultimately, it is expected that the insights gained will inform how existing and hypothetical frameworks can be most efficiently screened for CO_2_ adsorption applications.

## Experimental section

### Materials and methods

The Fe-BTT^13^ and Cu-BTT^12^ frameworks were synthesized as previously reported.

### Synthesis

#### Cr-BTT

Anhydrous chromium(ii) chloride (1.1 g, 9.0 mmol), 1,4-H_3_BTT (0.71 g, 3.6 mmol), DMF (300 mL) and triflic acid (3.2 mL 3.6 mmol) were added to a 500 mL Schlenk flask. The reaction mixture was heated at 393 K and stirred for 72 h to afford a light brown precipitate. The solid was collected by filtration and washed with 100 mL of DMF to yield 2.0 g (91%) of Cr-BTT. A sample of this compound (1.9 g, 3.3 mmol) was soaked in 100 mL of DMF at 393 K for 24 h after which the solvent was decanted, and the solid was then soaked in 100 mL of methanol at 343 K for 24 h. The methanol exchange was repeated three times, and the solid was collected by filtration to yield 1.3 g (87%) of Cr-BTT MeOH as a tan powder. A sample of this compound was fully desolvated by heating under dynamic vacuum (<10 μbar) at 433 K for 24 h to yield Cr-BTT as a light tan powder.

#### Mn-BTT

The synthesis of Mn-BTT was carried out according to the previously published procedure, with slight modification.^11^ In order to produce large quantities of the material for neutron diffraction experiments, the synthesis was scaled to a 500 mL autoclave bottle. Additionally, in order to maximize surface area, solvent was removed by decanting rather than filtration prior to activation.

It is important to mention that the M-BTT compounds in activated form can sometimes detonate upon exposure to air. As such, these materials should be handled with caution and in small quantities.

### Adsorption measurements

Ultra-high purity-grade (99.999% purity) helium, nitrogen, and carbon dioxide were used for all gas adsorption isotherm measurements. Adsorption data were collected at pressures ranging from 0 to 1.1 bar using a commercial Micromeritics ASAP 2020.^39^ Samples were transferred under a N_2_ atmosphere to preweighed analysis tubes that were capped with a Transeal. The samples were first evacuated on the activation station until the outgas rate was less than 3 mbar per min. The evacuated analysis tubes and samples were then carefully transferred to an electronic balance and weighed to determine the mass of the sample (typically 100–200 mg) after activation. For cryogenic measurements, the tube was immersed in liquid N_2_, covered with an isothermal jacket, and transferred back to the analysis port of the gas adsorption instrument. The outgas rate was again confirmed to be less than 3 mbar per min. Langmuir surface areas and pore volumes were determined from N_2_ adsorption isotherms measured in a 77 K liquid N_2_ bath, and were calculated using the Micromeritics software assuming a value of 16.2 Å^2^ for the molecular cross-sectional area of N_2_. For CO_2_ adsorption measurements, data were collected at temperatures ranging from 25 to 45 °C using a Micromeritics recirculating dewar connected to a Julabo F32-MC isothermal bath.

### Structural analysis

High-resolution powder neutron diffraction experiments were carried out on M-BTT (M = Cr, Mn, Fe, Cu) using BT1 at the National Institute of Standards and Technology (NIST) Center for Neutron Research (NCNR). All measurements were carried out on activated samples of ∼0.8 g. At NIST, samples were activated while heating under dynamic vacuum and then transferred into a He purged glove-box, loaded into a vanadium can equipped with a gas loading valve, and sealed using an indium O-ring. Powder neutron diffraction data were collected using a Ge (311) monochromator with an in-pile 60 collimator corresponding to a wavelength of 2.0780 Å. The samples were loaded onto a closed cycle refrigerator and then data were collected at 10 K. After data collection on the activated framework, CO_2_ was then loaded into each framework by first warming the samples to room temperature and then exposing them to a pre-determined amount of gas. Upon reaching an equilibrium pressure at the loading temperature, the sample was then slowly cooled (at a rate of 1 K min^–1^) to ensure complete adsorption of the CO_2_, and data was then collected again at 10 K. It should be noted that the activation procedure for Mn-BTT was not successful in extracting all of the solvent molecules from the material. This is consistent with previous reports, which reveal that solvent remains coordinated to 83% of the open metal sites after activation.^11^ Considering the low occupancy of CO_2_ at the Mn^2+^ sites and high occupancy of solvent, *in situ* experiments were not pursued for this sample.

Additional high-resolution neutron diffraction data were collected on the C2 diffractometer at Chalk River Laboratories at the Canadian Neutron Beam Center using a 1.2 g sample of Cr-BTT. The sample preparation and data collection of the bare and CO_2_-loaded framework were carried out using procedures similar to those described above. A wavelength of 2.3704 Å was used to collect data from ∼2.8° to 117° in 2*θ*. For this experiment, the gas loading apparatus designed for the top-loading, closed cycle refrigerator was not equipped with line heaters, and, because of this, cold zones caused the CO_2_ to freeze in the gas line before complete adsorption could be achieved. As such, loadings higher than 0.5 CO_2_ per Cr metal were not achieved and data were recollected at NIST. However, we note that results from the refinements of the bare structure and low CO_2_ loading are similar to those obtained at NIST (see ESI, Tables S7, S8 and Fig. S22†).

Powder neutron diffraction data were analyzed using the Rietveld method, as implemented in EXPGUI/GSAS.^40^,^41^ The M-BTT models were based on those previously determined,^12^,^13^,^15^ with the scale-factor and unit cell allowed to vary. Fourier difference methods were employed to locate residual solvent, extra-framework cations, and the adsorbed CO_2_ molecules in the structures of the activated bare materials.

X-ray diffraction data were also collected on Fe-BTT on 17-BM-B at the Advanced Photon Source at Argonne National Laboratory. The unactivated sample was loaded into a 1.0 mm borosilicate capillary that was then placed in a custom designed gas cell. The capillary was mounted onto the goniometer head at 17-BM and centered in the beam. The sample was then placed under dynamic vacuum, inserted in a N_2_ stream that was slowly heated to 150 °C where it was held for 2 h. Post-activation, the sample was gradually cooled to room temperature and then dosed with a small amount of He exchange gas. The sample was then further cooled to 100 K at a rate of 2 K min^–1^ where it was held for 30 min prior to data measurement to allow for temperature equilibration. The data were collected using a Si (111) monochromator (*λ* = 0.61072 Å, Δ*E*/*E* = 1.5 × 10^–4^). It should be noted that while efforts to activate the sample *in situ* succeeded in removing some of the methanol from the framework channels, they were unsuccessful at removing any of the methanol coordinated to the metal sites. Rietveld analysis was carried out on the data to elucidate the positions of extra-framework cations. Due to the inability to completely desolvated the framework at the synchrotron, data collection on the CO_2_-adsorbed samples were not pursued.

### Periodic density functional theory calculations

Periodic density functional theory (DFT) calculations were employed to compute first-principles CO_2_ binding energies using the Vienna Ab initio Simulation Package (VASP).^42^ To correctly model noncovalent interactions between CO_2_ and the framework, the dispersion corrected functional rev-vdW-DF2 + U was employed.^43^,^44^ The Hubbard *U* parameter is needed to model valence d electrons during adsorption and has been shown to be particularly important for frameworks with open-metal sites.^45^,^46^ The Hubbard *U* values in this work were based on values determined for the M_2_(dobdc) series: 6.1, 6.5, and 10.4 eV for Cr, Fe, and Cu, respectively.^47^ Based on the results of Poloni *et al.*,^35^ all metal centres are treated as high spin with antiferromagnetic ordering between metal centers. A plane wave basis set with a cutoff of 800 eV and PAW pseudopotentials were used.^48^ Atomic positions and lattice constants were optimized for bare frameworks without CO_2_ with forces on each atom converged to 2 × 10^–2^ eV Å^–1^. The atomic positions and lattice constants of the bare frameworks were optimized starting from the experimentally refined structures, and extra-framework cations were incorporated in order to balance the anionic framework charge. For Cu-BTT, the DFT relaxation included the experimentally observed Cu^+^ extra-framework cation which has a Cl and water molecule coordinated to it (described below in more detail, shown in Fig. 3 and S23†). For Cr-BTT and Fe-BTT, sodium cations were included in the tetrazolate cages, which corresponds more closely to their experimentally observed cation positions (shown in Fig. S21†). The placement of extra-framework cations in this work benefits from new diffraction data while previous studies by Poloni *et al.*^35^,^49^ placed the sodium cations coordinated to nitrogen atoms on two adjacent tetrazole ligands. Geometry optimizations of the bare framework allowed for the relaxation of all atoms, both in the framework and extra-framework cations, and the lattice constants. After relaxing the framework structure, the positions of CO_2_ molecules were relaxed starting from their positions in the experimental structure keeping the framework atoms fixed using the so-called rigid framework approximation. The CO_2_ binding enthalpies were calculated using harmonic vibrational frequencies (obtained for CO_2_ and the corresponding open metal site). Atomic positions for bound and unbound CO_2_ were optimized at the same level of theory as the framework. Point charges were assigned to framework atoms using the REPEAT scheme and the electrostatic potential generated by DFT for use in our simulations.^50^

### Grand canonical Monte Carlo simulations

Grand Canonical Monte Carlo (GCMC) simulations were performed using the RASPA molecular simulation software^51^ to compute room temperature CO_2_ and 77 K N_2_ isotherms. Total cycle numbers of 100 000 and 20 000 (evenly split between equilibrium and sampling cycles) were employed for the room temperature CO_2_ isotherms and 77 K N_2_ isotherms, respectively. Interactions between CO_2_ molecules and between N_2_ molecules were modelled with the TraPPE force field.^52^ Framework atoms were assigned interaction parameters using the Universal Force Field (UFF), and CO_2_-framework interactions were determined using Lorentz–Berthelot mixing rules.^53^

## Results and discussion

### CO_2_ adsorption properties

Low-pressure CO_2_ adsorption isotherms were collected for M-BTT (M = Cr, Mn, Fe, Cu) at 298, 308, and 318 K. At 298 K and pressures below 0.1 bar, the isotherms exhibit a steep initial rise that is indicative of highly polarizing adsorption sites, a phenomenon often observed for frameworks with coordinatively-unsaturated metals.^10^,^54^–^56^ The adsorption isotherm for Fe-BTT is the steepest of all the analogues, indicating a strong interaction with CO_2_ at low surface coverage, which is an area of interest for post-combustion flue gas capture.

To gain a quantitative estimation of binding strength, we calculated isosteric heats (*Q*_st_) of CO_2_ adsorption, which afford a measure of the average binding energy at constant coverage and can be determined by fitting the adsorption isotherms with a dual-site Langmuir–Freundlich equation (Fig. S3–S6†). The calculated zero-coverage isosteric heats (Table 1) range from 30.7 kJ mol^–1^ to 51.2 kJ mol^–1^ with the trend Fe > Mn > Cr > Cu. Consistent with previous studies of the M_2_(dobdc) (M = Mg, Mn, Fe, Co, Cu, Zn) frameworks, the observed isosteric heats cannot be rationalized based upon ionic radii alone.^10^,^57^ A computational study of the M_2_(dobdc) series by Yu *et al.* revealed that the electrostatic interactions are likely dictated by nuclear screening effects that cause variations in the effective charge experienced by the CO_2_ at the open metal site.^57^ Computational work conducted both in this study on M-BTT frameworks (M = Cr, Fe, Cu) and an earlier work by Poloni *et al.* supports the experimentally observed trend in binding energy (Fe > Cr > Cu).^35^

**Table 1:** Experimental and computed data for CO_2_ adsorbed at site I in M-BTT.^*a*^ Distances and angles are listed in Å and degrees, respectively

M^2+^	Isotherm		DFT (rev-vdW-DF2 + U)				Diffraction data		
	SA (m^2^ g^–1^)	*Q*_st_ (kJ mol^–1^)	–*H*_b_ (kJ mol^–1^)	M–O(CO_2_)^*b*^	N···C^*c*^	O–C–O^*d*^	M–O(CO_2_)^*b*^	N···C^*c*^	O–C–O^*d*^
Cr	1820	36.7	36.6	2.625	2.914	176.7	2.66(4)	3.04(2)	176(2)
Mn^*e*^	2050	45.6	—	—	—	—	—	—	—
Fe	1700	51.2	51.7	2.301	3.019	174.4	2.36(3)	3.00(3)	180(4)
Cu	1700	30.7	29.4	2.567	3.041	177.1	2.60(3)	3.01(1)	177(1)

^*a*^Experimentally-determined surface areas (SA), zero-coverage isosteric heats of adsorption (*Q*_st_), and select geometric parameters for surface bound CO_2_ are presented; computed enthalpies of adsorption (–*H*_b_) and select geometric parameters are included for comparison. Values in parentheses indicate one standard deviation.

^*b*^Distance from the metal center to the bound oxygen of CO_2_.

^*c*^Distance between carbon of bound CO_2_ and the nearest tetrazole nitrogen.

^*d*^Intramolecular angle of bound CO_2_.

^*e*^Due to the challenge of removing all solvent molecules bound to the open Mn^2+^ sites, data collection using neutron powder diffraction and DFT calculations on Mn-BTT were not pursued.

**Fig. 2:**
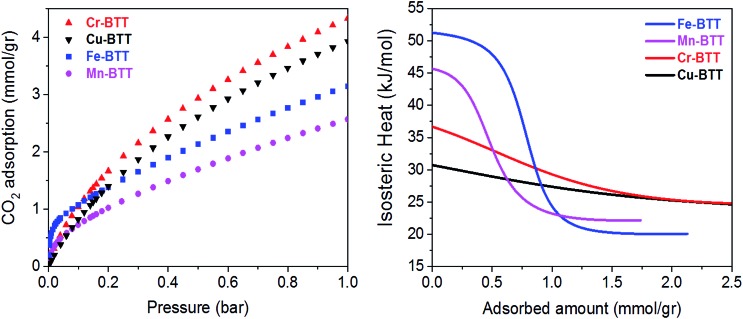
(Left) Excess CO_2_ adsorption isotherms obtained at 298 K for various M-BTT analogs. (Right) Isosteric heats of adsorption, *Q*_st_, plotted as a function of CO_2_ loading.

Upon increasing the CO_2_ loading, a rapid drop is observed in the isosteric heat for Fe- and Mn-BTT, likely indicating that a portion of the primary adsorption sites are blocked by residual solvent molecules. Indeed, previous *in situ* structural studies of D_2_ adsorption in this series of frameworks revealed a significant number of metal sites are blocked by solvent molecules that cannot be removed using standard activation procedures.^11^,^13^ Rietveld refinement of the neutron diffraction data collected in this study on the bare Cr-BTT, Fe-BTT, and Cu-BTT frameworks post-activation revealed that ∼23%, ∼65%, and ∼17% of the open metal sites, respectively, are blocked by coordinated solvent. Therefore, the overall trend for the number of available open metal sites follows the order Cu ≈ Cr > Fe > Mn, consistent with what has been previously reported.^11^,^13^,^15^ The smaller number of available open metal sites in Mn-BTT causes the isosteric heat to drop faster than that of the Fe-analog (Fig. 2), while the larger number of available open metal sites in Cu-BTT and Cr-BTT can be used to rationalize their higher overall capacity for CO_2_ at 1 bar, despite their lower zero-coverage isosteric heat of CO_2_ adsorption (Table 1).

### Characterization of multi-site CO_2_ adsorption in M-BTT

**Fig. 3:**
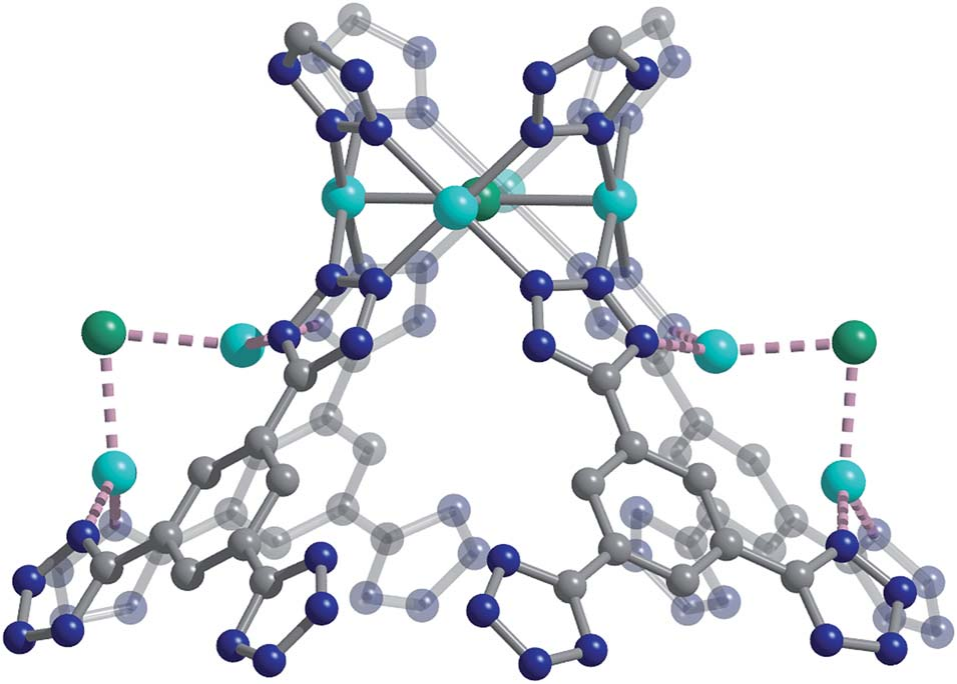
High-resolution neutron powder diffraction structure of Cu-BTT showing the extra-framework Cu^+^ cations that bridge N atoms of the tetrazolate ligand. Cyan, grey, blue, and green spheres represent Cu, C, N, and Cl atoms, respectively; H atoms are omitted for clarity.

We used high-resolution neutron diffraction to elucidate the CO_2_ adsorption behavior of the M-BTT frameworks, excluding Mn-BTT, due to the difficulty of removing a sufficient amount of coordinated solvent from the metal sites. With the general chemical formula [(M_4_Cl)_3_(BTT)_8_]^3–^, M-BTT requires extra-framework metal cations to balance anionic charge. Although the positions of the extra-framework cations for Cr-BTT could not be determined due to a low coherent scattering neutron cross section, Cu cations were found in a slightly distorted trigonal planar environment coordinated by two N atoms of the tetrazolate ring (1.97(3) Å) and a Cl^–^ (2.51(6) Å) (Fig. 3). This coordination geometry is indicative of the presence of a reduced Cu^+^ ion with an occupancy that charge balances the Cu-BTT framework to suggest an overall composition of Cu_6_[(Cu_4_Cl)_3_(BTT)_8_]Cl_3_ for fully activated Cu-BTT.^58^,^59^ For Fe-BTT, the extra-framework cations are found directly above the Cl-centered [Fe_4_Cl]^7+^ cluster at a Cl···Fe distance of 4.4(1) Å (Fig. S21†). A small amount of excess scattering density was also observed in the axial position of the Cu^2+^ cations found in the [Cu_4_Cl]^7+^ cluster of Cu-BTT, supporting the evidence for incomplete activation noted above. In this case, the excess scattering density appears to be a single atom, likely due to water adsorption, and hence is modelled as a single oxygen at an occupancy of 0.17(2) and a distance of 2.17(6) Å from the Cu^2+^ site. A similar procedure was carried out to model excess scattering density in Cr- and Fe-BTT (Tables S6 and S10†).

Following structure determination of the activated materials, the samples were dosed with ∼0.5 CO_2_ per framework M^2+^ and the analysis was repeated. For all analogs, the primary adsorption site consists of CO_2_ bound in an end-on configuration at the M^2+^ cation (Fig. 1), confirming the presence of strong electrostatic interactions indicated by the high initial isosteric heats of adsorption. The M^2+^–O(CO_2_) distances range from 2.36(3) Å for Fe-BTT to 2.60(3) Å for Cu-BTT. While Cr-BTT binds CO_2_ more strongly than Cu-BTT, the similar M^2+^–O(CO_2_) distances in both analogs can be rationalized based on the larger ionic radii of Cr compared to Cu.

**Fig. 4:**
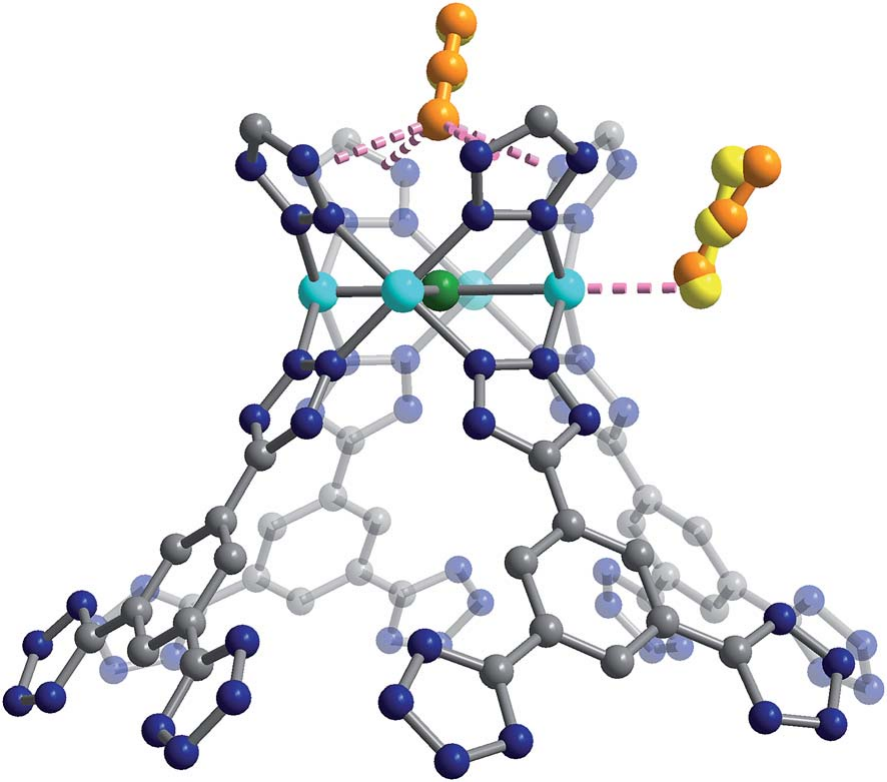
Structure of Cu-BTT showing the primary (site I, right) and secondary (site II, top) CO_2_ adsorption sites, as identified by *in situ* powder neutron diffraction and DFT calculations (yellow and orange ball-and-stick representations, respectively). Cyan, grey, blue, and green spheres represent Cu, C, N, and Cl atoms, respectively; H atoms are omitted for clarity.

Secondary van der Waals interactions between the CO_2_ carbon atoms and the nitrogen atoms on the nearest tetrazole ring in each framework (located at a C···N distance of ∼3 Å) lead to M–O–C(CO_2_) angles ranging from 106(1)° for Cu-BTT to 116(2)° for Fe-BTT. DFT angles are overestimated for this parameter at 127.5° for Cu-BTT and 134.0° for Fe-BTT, but the trend between the metals is consistent with the experiment. Fe-BTT has the strongest binding energy and the largest angle. The computed angle for Cr-BTT is 126.3°, so Cr and Cu have similar binding angles despite Cr having a stronger binding energy; however, in the case of Cu, the presence of an extra-framework cation in the main adsorption cage could also influence the CO_2_ orientation. The intramolecular CO_2_ angles show minimal deviation from the expected linear geometry. While these angles are in good agreement with DFT calculations (Table 1) and indicate that there is little electronic activation of the bound CO_2_ molecule, nothing can be said about the trend in the CO_2_ angles due to the error associated with the diffraction experiment. The binding enthalpies and geometries of CO_2_ in M-BTT (M = Cr, Fe, Cu) were also determined by periodic DFT calculations, and the results are presented in Tables 1 and 2. The theoretical site I CO_2_ binding energies (Table 1) are in excellent agreement with experimentally-determined zero-coverage isosteric heats of adsorption, importantly capturing the observed experimental trend. The theoretical M–O(CO_2_) distances for site I (Table 1) in the Cr- and Cu-BTT frameworks are also in near perfect agreement with the experiment (differing by < 0.04 Å) and within the range of the standard deviation (Fig. 4 and S20†). The theoretical site I M–O(CO_2_) distance in Fe-BTT is also in excellent agreement with experiment (differing by < 0.06 Å). When considering the margin of error associated with the diffraction experiment (≈0.03–0.04 Å), there is almost no difference between the computationally and experimentally determined metal–oxygen distances for CO_2_ binding in Cr-, Fe-, or Cu-BTT. We note that the calculated M–O(CO_2_) distances are also in much better agreement with experiment than those previously predicted for the M-BTT series,^35^ which show deviations up to ∼0.2 Å (the values reported previously use a different flavour of DFT and different positions for extra-framework cations). While the calculated trend in M–O(CO_2_) binding distances (Cr ≈ Cu > Fe) presented here corresponds with the one observed experimentally, the Cr–O(CO_2_) bond distance is slightly longer than the Cu–O(CO_2_) distance despite the stronger Cr–CO_2_ binding energy. This is likely due to the aforementioned difference in the ionic radii of Cu^2+^ and Cr^2+^. Note that previous work by Poloni *et al.* reported that this distance correlated with binding strength; however, their calculated Cu–O(CO_2_) distance was only 0.005 Å longer than Cr–O(CO_2_) and therefore is also in good agreement with experiment.

**Table 2:** Experimental and computed binding enthalpies and geometries (DFT) for site II CO_2_. Distances and angles are listed in Å and degrees, respectively

M^2+^	DFT (rev-vdW-DF2 + U)			Diffraction data	
	–*H*_b_ (kJ mol^–1^)	O···Cl^*a*^	O–C–O^*b*^	O···Cl^*a*^	O–C–O^*b*^
Cr	26.6	3.303	179.1	3.17(3)	179(6)
Fe	24.5	3.237	179.7	3.38(2)	179(7)
Cu	26.8	3.300	179.4	3.27(2)	179(8)

^*a*^Theoretical and computed bond distances describe the distance between the oxygen atom in CO_2_ and the chlorine atom in the center of the cluster.

^*b*^The intramolecular O–C–O angle of the CO_2_ molecule.

Upon increasing the CO_2_ loading from 0.5 to 1.5 CO_2_ per M^2+^, three additional, weaker adsorption sites become populated (Fig. 1). In the case of Mn- and Fe-BTT, the significant drop in their isosteric heats of CO_2_ adsorption (Fig. 2. (right)) indicates that preference for site I persists to room temperature. In contrast, the relatively flat nature of the isosteric heats of adsorption for Cr- and Cu-BTT implies similar binding energies for the different binding sites at room temperature, an observation that is supported by the predicted binding enthalpies of sites I and II determined *via* DFT calculations (Tables 1 and 2). A comparison of the DFT-derived CO_2_ binding energies for sites I and II provides further valuable insight into the behavior of the framework adsorption isotherms. While the binding energy of CO_2_ at site I depends strongly on metal identity, the binding strength at site II appears to be nearly independent of the metal. Indeed, the DFT binding strengths of CO_2_ at site II (Table 2) vary by only 2 kJ mol^–1^ across the frameworks studied. Given that the M-BTT frameworks are isostructural, it is perhaps unsurprising that CO_2_ should adsorb with similar binding energies at secondary adsorption sites. The DFT results are further supported by the similar isosteric heat values extracted for the frameworks at higher surface coverage (Fig. 2).

The secondary CO_2_ adsorption site in Cu-BTT (Fig. 1) is rotationally disordered above the [Cu_4_Cl]^7–^ cluster. The closest oxygen atom of carbon dioxide bound at this site is approximately 3.27 Å from the Cl^–^ and 3.44 Å from the center of the tetrazolate ring, distances that are in agreement with what is expected based on the van der Waals radii of oxygen and chlorine. Interestingly, the extra-framework cations in Fe-BTT also reside just above the Cl^–^ anion in the [M_4_Cl]^7–^ cluster and partially block the secondary adsorption site in this structure. In addition to the blocking of a large number of primary adsorption sites due to solvent molecules, the positioning of this cation may also influence the significant drop observed in the *Q*_st_ for Fe-BTT relative to Cr- and Cu-BTT. As was the case for CO_2_ bound at site I, the DFT calculated binding geometries for CO_2_ at site II match well with the experimentally-determined values (Table 2). The largest deviation was observed for Cr-BTT, for which the DFT calculations revealed a Cl···O(CO_2_) distance that is ∼0.1 Å longer than that observed experimentally. The experimentally and theoretically determined structures for Cu-BTT are overlaid in Fig. 4 (see also Fig. S20†).

At site III, CO_2_ is rotationally disordered between two carbon atoms of the BTT^3–^ linker and located at a C···O distance of ∼3.27 Å from the framework wall. Although this site is twice as abundant as site II, the adsorption of CO_2_ at site III is expected to be weaker given its lower occupation observed *via in situ* neutron diffraction. It should be noted that while the CO_2_ molecules located at sites III and IV are somewhat disordered, the intermolecular CO_2_ distances for these two sites are shorter than those of the closest CO_2_-framework distances. It may be that the population of site II helps to stabilize the population of these two additional adsorption sites, an observation that is supported by a pronounced plateau in *Q*_st_ at higher loadings (Fig. 2). It is also possible that CO_2_ molecules located at sites III and IV afford additional stabilization to CO_2_ molecules located at site II (Fig. 1), in a manner similar to that suggested in an earlier study on CH_4_ binding in the metal–organic framework Cu_3_(btc)_2_.^60^

Positional disorder in the CO_2_ molecules bound at sites III and IV precluded periodic DFT analysis, and therefore we turned to force fields to quantify any stabilizing influence of neighboring CO_2_ molecules on the adsorbed gas at these positions. For this analysis, we arranged the site III and IV CO_2_ molecules at their experimental unit cell positions around site II CO_2_ with the DFT-determined binding geometry. We found the strength of the interaction between site II and site III CO_2_ molecules to be –4.58, –3.13, and –3.26 kJ mol^–1^ for Cu-, Cr-, and Fe-BTT, respectively. Interactions between CO_2_ located at site II and site IV were found to be smaller, although of the same order of magnitude. Considering the magnitude of the CO_2_ binding enthalpies at site II (Table 2), it is expected that interactions with CO_2_ molecules at sites III and IV are unlikely to have a large impact on the site II binding enthalpy. However, the CO_2_ molecules at sites III and IV exhibit weaker interactions with the framework compared to that of site II and may be primarily stabilized by interactions with the closest site II molecule.

Because the strength of CO_2_ binding at site I is dependent on the identity of the framework metal and the binding strength at site II is not, the theoretical difference between the site I and site II binding energies follows the same trend across the M-BTT series as the site I binding energy (Fe > Cr > Cu). DFT calculations show that CO_2_ binds more strongly at site I than site II in Fe-, Cr-, and Cu-BTT by 27.2, ∼10, and 2.6 kJ mol^–1^, respectively. The higher binding strength at site I is supported by the higher experimentally observed site I occupancy at low CO_2_ loadings in diffraction experiments. We note that while an energy difference as small as ∼3 kJ mol^–1^ is enough to distinguish sites I and II and lead to sequential occupation of adsorption sites at 10 K, at room temperature there is enough thermal energy such that there is no strongly preferred adsorption site. A similar phenomenon has been observed for CH_4_ binding in Cu_3_(btc)_2_ using powder neutron diffraction, wherein thermodynamic preference for a given binding site at 150 K is lost at higher temperatures.^60^ Room-temperature CO_2_ adsorption isotherms for Cr-, Fe- and Cu-BTT plotted on semi-log and logarithmic axes are shown in Fig. S18 and S19.† On a semi-log scale, Cr- and Cu-BTT exhibit Langmuir-type CO_2_ adsorption behavior, while in Fe-BTT CO_2_ appears to bind most strongly at low pressures. This latter observation is supported by DFT calculations, which predict the largest difference in site I and II binding energies for Fe-BTT. The combined theoretical and experimental work presented here suggests that differences in binding energy of >20 kJ mol^–1^ may be needed to observe a truly sequential loading of different binding sites in room temperature adsorption isotherms.

### Comparison with previous computational work

It is of interest to compare the DFT results obtained here to previous computational work by Poloni *et al.*, which examined CO_2_ adsorption at the primary site in M-BTT for both existing and hypothetical analogs.^35^ The results in the previous work^35^ were obtained using atomic orbital basis sets and a PBE + D2 + U van der Waals corrected functional, whereas this work used a plane wave basis set and the rev-vdW-DF2 + U functional. Additionally, the two studies differ in the placement of the extra-framework cations. Both of these factors will contribute to differences in the geometric parameters and binding energies reported in the two studies; however, overall trends can be examined.

In particular, the site I binding energies and trend in M–O(CO_2_) distances reported previously are in good agreement both with those in this work and with the experimental isosteric heats of adsorption. This supports the conclusion that the interaction with the M^2+^ cation dominates and this site is not particularly sensitive to the position of the framework cation. On the other hand, the site I binding geometries from this study more closely match the diffraction results. For example, the M–O(CO_2_) binding distances at the primary adsorption sites were on average 0.045 Å closer to the experimental values compared to those reported previously. Our distances are slightly shorter than experiment while those of Poloni *et al.* are slightly longer. This could be due to the fact that the rev-vdW-DF2 functional was reparameterized specifically to improve bond distances while maintaining good binding energies; however, functional dependence in this system has not been systematically examined. The previous study also observed that stronger CO_2_ binding correlated with deviations from the expected linear geometry of CO_2_ and shorter distances between the tetrazole nitrogen and the CO_2_ carbon. In the diffraction results presented here, neither of these trends is observed. Although our DFT calculations started from the experimental structure, we observe the same trend in CO_2_ bending as Poloni *et al.* Moreover, the distance between the tetrazole nitrogen and CO_2_ carbon shows no clear dependence on the binding energy and the trend between the metals is different in the experiment (Cr > Cu > Fe), our calculations (Cu > Fe > Cr), and those of Poloni *et al.* (Cu > Cr > Fe). However, both experiment and Poloni *et al.* report the shortest N–C distance for the strongest binding metal. Additionally, in our calculations for Fe-BTT sodium migrated closer to Cl than the experimentally observed Fe cation. It is possible that the location and identity of the extra framework Fe cations has an impact on the CO_2_ binding geometry.

The diffraction data presented in this work has created the opportunity for a future benchmarking study to determine the influence of different DFT functionals on the accurate prediction of CO_2_ adsorption properties in the M-BTT framework series. Relative differences between Cr- and Fe-BTT structures computed in this work compared to the previous study may have to do with the placement of sodium cations, the respective choices of functional, and the choices in the level-of-theory. Furthermore, the specific identity of the extra-framework cations may play an important role in counterbalancing intra-framework forces. For example, DFT studies exploring whether extra-framework cations with different ionic radii and partial charge could occupy different sites based on steric effects, which has been suggested in the literature,^61^ have not been performed.

## Conclusions

We have demonstrated that *in situ* neutron diffraction can provide insights into how to optimize existing metal–organic frameworks for CO_2_ adsorption (*e.g.*, *via* metal substitution) while also providing detailed structural information that can be used to validate computational methods aimed at predicting the behavior of metal–organic frameworks with open metal sites. Indeed, in this study, calculated CO_2_ binding energies and geometries were in good agreement with the experimental results, and such validation of computational approaches for adsorption applications opens the door to predicting the behavior of synthesized and even hypothetical metal–organic frameworks.

The foregoing results also provide experimental evidence that the strength of adsorption at the open metal site in the M-BTT series can be rationalized based on metal identity, including the partial charge on the electropositive M^2+^ cations. This observation is confirmed by both prior computational work and the results of DFT calculations presented here.^35^ DFT calculations also suggest that the heat of adsorption at site II is not dependent on metal identity. Strong CO_2_ adsorbents, like Fe-BTT, display sequential adsorption behaviour, which could potentially be exploited for applications such as CO_2_ conversion. This study shows the need for combined experimental and computational studies capable of predicting framework properties, a process that might eventually allow the rapid identification of target materials for CO_2_ capture, as well as a host of other applications.

## Conflicts of interest

There are no conflicts to declare.

## Supplementary Material

Supplementary information

Supplementary information

Crystal structure data
