# Supplementary material for: An experimental and computational study of CO2 adsorption in the sodalite-type M-BTT (M = Cr, Mn, Fe, Cu) metal–organic frameworks featuring open metal sites
Source: Chem Sci. 2018 Apr 23;9(20):4579–88. doi: 10.1039/c8sc00971f (PMC5969499; doi:10.1039/c8sc00971f)
Supplement: Supplementary file 2 [file SC-009-C8SC00971F-s002.pdf]

Electronic Supplementary Material (ESI) for Chemical Science.
This journal is © The Royal Society of Chemistry 2018

Supporting Information for:

An experimental and computational study of CO_2 adsorption in the sodalite-type M-BTT (M = Cr, Mn, Fe, Cu) metal–organic frameworks featuring open metal sites†

M. Asgari[‡], S. Jawahery[‡], E. D. Bloch, M. R. Hudson, R. Flacau, B. Vlaisavljevich, J. R. Long, C. M. Brown, and W. L. Queen*

Table of Contents

Gas adsorption measurements 5

Figure S1. Nitrogen adsorption isotherms for M-BTT analogues collected at 77 K. 5

Table S1. Surface areas for the M-BTT frameworks. 7

Figure S2. Nitrogen adsorption of M-BTT analogues at 298 K. 7

Fitting M-BTT CO_2 adsorption isotherms 7

Figure S3. Dual-site Langmuir fits for CO_2 adsorption in Fe-BTT at 298, 308, and 318 K. 8

Figure S4. Dual-site Langmuir fits for CO_2 adsorption in Mn-BTT at 298, 308, and 318 K. 8

Figure S5. Dual-site Langmuir fits for CO_2 adsorption in Cr-BTT at 298, 308, and 318 K. 9

Figure S6. Dual-site Langmuir fits for CO_2 adsorption in Cu-BTT at 298, 308, and 318 K. 9

Table S2. Dual-site Langmuir parameters for CO_2 adsorption in M-BTT. 10

Calculating Isosteric heats of adsorption 10

Figure S7. Neutron powder diffraction data collected on an activated sample of Cu-BTT at 10 K. The green line, crosses, and red line represent the background, experimental, and calculated diffraction patterns, respectively. The blue line represents the difference between experimental and calculated patterns. 11

Figure S8. Neutron powder diffraction data collected at 10 K for a sample of Cu-BTT loaded with 0.533 CO_2 per Cu site (6.396 CO_2 per unit cell). The green line, crosses, and red line represent the background, experimental, and calculated diffraction patterns, respectively. The blue line represents the difference between experimental and calculated patterns. 12

Table S4. Fractional atomic coordinates, occupancies, and isotropic displacement parameters obtained from Rietveld refinement of the structural model for Cu-BTT (loaded with 0.533 CO_2 per Cu site) against diffraction data presented in Figure S8. The structure is cubic with Pm-3m space group, a = 18.5295(3) Å, and V = 6362.0(3) Å^3. Values in parentheses represent standard deviation. GOF parameters: χ^2= 1.01, wRp = 0.0354, Rp = 0.0305. (The number for corresponding CIF file in CCDC database: 1582006) 12

Figure S9. Neutron powder diffraction data collected at 10 K on a sample of Cu-BTT loaded with 1.558 CO2 per Cu site (18.696 CO_2 per unit cell). The green line, crosses, and red line represent the background, experimental, and calculated diffraction patterns, respectively. The blue line represents the difference between experimental and calculated patterns. 13

Figure S10. Neutron powder diffraction data collected at 10 K for an activated sample of Cr-BTT. The green line, crosses, and red line represent the background, experimental, and calculated diffraction patterns, respectively. The blue line represents the difference between experimental and calculated patterns. 15

Table S6. Fractional atomic coordinates, occupancies, and isotropic displacement parameters obtained from Rietveld refinement of the activated Cr-BTT structural model against data presented in Figure S10. The structure is cubic, space group Pm-3m, a = 18.6689(4) Å, and V = 6506.6(4) Å^3. Values in parentheses indicate one standard deviation in the refined value. GOF parameters: χ^2 = 1.06, wRp = 0.0296, Rp = 0.0255. (The number for corresponding CIF file in CCDC database: 1582001). 15

Figure S11. Neutron powder diffraction data collected at 10 K on a sample of Cr-BTT loaded with 0.56 CO_2 per Cr site (6.72 CO_2 per unit cell). The green line, crosses, and red line represent the background, experimental, and calculated diffraction patterns, respectively.

The blue line represents the difference between experimental and calculated patterns. Data was collected at NIST. 16

Table S7. Fractional atomic coordinates, occupancies, and isotropic displacement parameters obtained from Rietveld refinement of the structural model for Cr-BTT loaded with 0.56 CO_2 per Cr site (6.72 CO_2 per unit cell) against the data presented in Figure S11. The structure is cubic, space group Pm-3m, a = 18.6867(5) Å, V = 6252.2(5) Å^3. Values in parentheses indicate standard deviation. GOF parameters: χ^2 = 1.16, wRp = 0.0333, Rp = 0.0288. (The number for corresponding CIF file in CCDC database: 1582003). 16

Figure S12. Neutron powder diffraction data collected at 10 K on Cr-BTT loaded with 0.436 CO_2 per Cr site (5.23 CO_2 per unit cell). The green line, crosses, and red line represent the background, experimental, and calculated diffraction patterns, respectively. The blue line represents the difference between experimental and calculated patterns. Data was collected at Chalk River Laboratories at the Canadian Neutron Beam Center. 17

Table S8. Fractional atomic coordinates, occupancies, and isotropic displacement parameters obtained from Rietveld refinement of the structural model for Cr-BTT loaded with 0.436 CO_2 per Cr site (5.23 CO_2 per unit cell) against the data presented in Figure S12. The structure is cubic, space group Pm-3m, a = 18.7761(6) Å, V = 6619.4(6) Å^3. Values in parentheses indicate standard deviation. GOF parameters: χ^2 = 2.13, wRp = 0.0360, Rp = 0.0284. (The number for corresponding CIF file in CCDC database: 1582004). 17

Figure S13. Neutron powder diffraction data collected at 10 K on Cr-BTT loaded with 1.275 CO_2 per Cr site (15.304 CO_2 per unit cell). The green line, crosses, and red line represent the background, experimental, and calculated diffraction patterns, respectively. The blue line represents the difference between experimental and calculated patterns. 18

Table S9. Fractional atomic coordinates, occupancies, and isotropic displacement parameters obtained from Rietveld refinement of the structural model for Cr-BTT loaded with 1.275 CO_2 per Cr site (15.304 CO_2 per unit cell) against data shown in Figure S13. The structure is cubic, space grupd Pm-3m, a =18.6816(3) Å, V = 6520.0(4) Å^3. Values in parentheses indicate standard deviation. GOF parameters: χ^2 = 1.18, wRp = 0.0317, Rp = 0.0271. (The number for corresponding CIF file in CCDC database: 1582002). 19

Figure S14. Neutron powder diffraction data obtained at 10 K on an activated sample of Fe-BTT. The green line, crosses, and red line represent the background, experimental, and calculated diffraction patterns, respectively. The blue line represents the difference between experimental and calculated patterns. 20

Table S10. Fractional atomic coordinates, occupancies, and isotropic displacement parameters obtained from Rietveld refinement of the activated Fe-BTT structural model against the data presented in Figure S14. The structure is cubic, space group Pm-3m, a =18.8957(4) Å, V = 6746.6(5) Å^3. Values in parentheses indicate standard deviation. GOF parameters: χ^2 = 1.3, wRp = 0.0272, Rp = 0.0236. (The number for corresponding CIF file in CCDC database: 1582010). 20

Figure 15. Synchrotron powder diffraction data obtained at 100 K on an activated sample of Fe-BTT. The green line, crosses, and red line represent the background, experimental, and calculated diffraction patterns, respectively. The blue line represents the difference between experimental and calculated patterns. 21

Table S11. Fractional atomic coordinates, occupancies, and isotropic displacement parameters obtained from Rietveld refinement of the activated Fe-BTT structural model against the data in Figure S15. The structure is cubic, space group Pm-3m, a = 18.8004(1) Å, V = 6645.1 (1) Å^3. Values in parentheses indicate one standard deviation in the refined value.

GOF parameters: χ^2 = 3.20, wRp = 0.0289, Rp = 0.0213. (The number for corresponding CIF file in CCDC database: 1582005). 21

Figure S16. Neutron powder diffraction data obtained at 10 K on a sample of Fe-BTT loaded with 0.662 CO_2 per Fe site (7.944 CO_2 per unit cell). The green line, crosses, and red line represent the background, experimental, and calculated diffraction patterns, respectively. The difference between experimental and calculated patterns is represented as a blue line. 22

Figure S17. Neutron powder diffraction data obtained at 10 K on a sample of Fe-BTT loaded with 1.716 CO_2 per Fe site (20.592 CO_2 per unit cell). The green line, crosses, and red line represent the background, experimental, and calculated diffraction patterns, respectively. The blue line represents the difference between experimental and calculated patterns. 24

Table S13. Fractional atomic coordinates, occupancies, and isotropic displacement parameters obtained from Rietveld refinement of the structural model for Fe-BTT loaded with 1.716 CO_2 per Fe site (20.592 CO_2 per unit cell) against the data presented in Figure S17. The structure is cubic, space group Pm-3m, a = 18.5952(4) Å, V = 6746.1(4) Å^3. Values in parentheses indicate standard deviation. GOF parameters: χ^2 = 1.56, wRp = 0.0302, Rp = 0.0267. (The number for corresponding CIF file in CCDC database: 1582008). 25

Figure S18. Semi-log scale plotted isotherm of CO_2 adsorption in M-BTT (M = Cr, Mn, Fe, and Cu). 26

Figure S19. Log scale plotted isotherm of CO_2 adsorption in M-BTT (M= Cr, Mn, Fe, and Cu). 26

Figure S20. Locations of the primary and secondary CO_2 adsorption sites in Cu-BTT as determined from DFT calculations (orange ball and stick representation) and *in situ* neutron powder diffraction (yellow ball-and-stick representation). Cyan, green, blue, and gray spheres represent framework Cu, Cl, N, and C atoms, respectively; H atoms are omitted for clarity. 27

Figure S21. The position of extra-framework Fe cations inside Fe-BTT. Orange, blue, and grey spheres represent Fe, N, and C atoms, respectively 27

Figure S22. Comparison of the location of primary CO_2 adsorption sites in Cr-BTT as determined from analysis of diffraction data collected at NIST (pink ball-and-stick representation) and at the Chalk River Laboratories at the Canadian Neutron Beam Center (yellow ball-and-stick representation). Green, blue, and grey spheres represent Cr, N, and C atoms, respectively; H atoms are omitted for clarity. 28

Figure S23. Extra-framework cationic clusters in Cu-BTT as determined from Rietveld analysis of high-resolution powder neutron diffraction data. Cyan, grey, blue, green and red spheres represent Cu, C, N, Cl and O atoms, respectively; H atoms are omitted for clarity. An extra-framework cationic cluster consists of two Cu and one Cl atom – waters are also observed to coordinate to neighboring Cu atoms. 28

References 29

Gas adsorption measurements

UHP-grade (99.999 % purity) helium, nitrogen, and carbon dioxide were used for all adsorption measurements. Gas adsorption isotherms for pressures in the range 0–1 bar were measured using a Micromeritics ASAP 2020 instrument. For standard measurements in ASAP low-pressure glass sample holders, activated samples were transferred under a N_2 atmosphere to pre-weighed analysis tubes, which were capped with a Transeal. The samples were evacuated on the ASAP until the outgas rate was less than 3 μbar/min. The evacuated analysis tubes containing degassed samples were then carefully transferred to an electronic balance and weighed to determine the mass of sample (typically 100–200 mg). For cryogenic measurements, the tube was fitted with an isothermal jacket and transferred back to the analysis port of the gas adsorption instrument. The outgas rate was again confirmed to be less than 3 μbar/min. BET surface areas and pore volumes were determined by measuring N_2 adsorption isotherms in a 77 K liquid N_2 bath and calculated, assuming a value of 16.2 Å^2 for the molecular cross-sectional area of N_2. Adsorption isotherms between 25 and 45°C were measured using a recirculating dewar (Micromeritics) connected to a Julabo F32-MC isothermal bath.

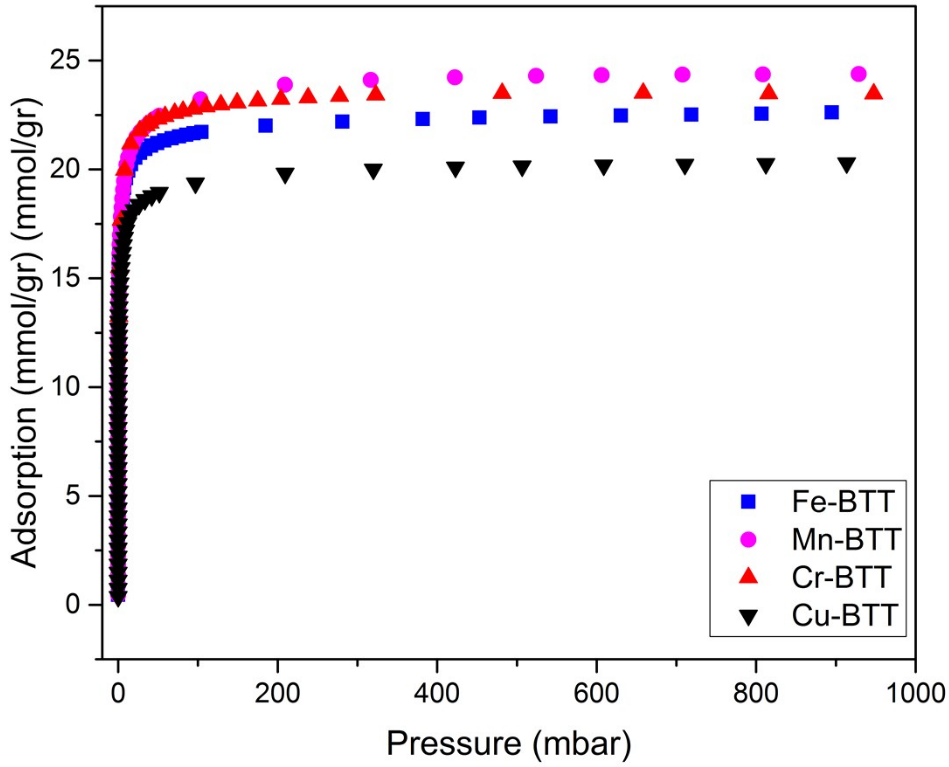

Figure S1. Nitrogen adsorption isotherms for M-BTT analogues collected at 77 K.

Pore Volume calculations based on the adsorption data for nitrogen at 77 K

As shown in Figure S1, the adsorption isotherm for N_2 at 77 K ceases to be a function of pressure when the pressure exceeds 200 mbar, implying that the structure has been saturated with nitrogen. Therefore, the uptake of N_2 at highest measured pressure can be considered as the full capacity of the framework for an inert small molecule, and the pore volume can then be calculated from the following formula:

$$Pore\ Volume = \frac{N_{N_2}^{Sat} * M_{wN_2}}{1000 * d_{liqN_2}\big|_{77K}}$$

Where $N_{N_2}^{Sat}$ is the the amount of nitrogen adsorbed at the highest pressure point in mmol/g, M_{wN_2} is the molecular weight dinitrogen in g/mol and $d_{liqN_2}\big|_{77K}$is the density of liquid nitrogen at 77 K in g/mL.

Therefore, for the M-BTT pore volumes we obtain:

$$Pore\ Volume_{Fe-BTT} = \frac{22.6 * 28.013}{1000 * 0.807} = 0.785\ mL/g$$
$$Pore\ Volume_{Mn-BTT} = \frac{24.4 * 28.013}{1000 * 0.807} = 0.846\ mL/g$$
$$Pore\ Volume_{Cr-BTT} = \frac{23.5 * 28.013}{1000 * 0.807} = 0.815\ mL/g$$
$$Pore\ Volume_{Cu-BTT} = \frac{23.5 * 28.013}{1000 * 0.807} = 0.704\ mL/g$$

Surface area calculations based on the N_2 adsorption data at 77 K

Based on the BET theory, we have the following equation:

$$\frac{1}{N[\left(\frac{p_0}{p}\right) - 1]} = \frac{c-1}{N_m c}\left(\frac{p}{p_0}\right) + \frac{1}{N_m c}$$

Where N is the amount of gas adsorbed in mmol/g, N_m is the monolayer adsorption in mmol/g, p is the partial pressure of N_2 and p_0 is the saturation pressure of N_2 at the measurement temperature.[1] A plot of $\frac{1}{N[\left(\frac{p_0}{p}\right) - 1]}$ versus $\frac{p}{p_0}$ and the best possible trendline is fit to the points on the plot. We note that points with low $\left(\frac{p}{p_0}\right)$ are chosen such that the *c* parameter stays positive and high R^2 values are obtained. With M as the slope and I as the intersect of the plot, N_m can be obtained from the following formula:

$$N_m = \frac{1}{M + I}$$

Thr surface area can then be obtained using the equation $SA = N_m \cdot N_A \cdot CSA_{N_2}$, where N_A is Avogadro's number and CSA_{N_2} is the cross sectional area of one N_2 molecule at 77 K (taken to be 16 Å^2). The above calculations were carried out on the nitrogen adsorption isotherm data collected at 77 K for M-BTT; the resulting surface areas are given in Table S1.

Table S1. Surface areas for the M-BTT frameworks.

Sample	Surface Area (m^2/g)
**Fe-BTT**	2044.8
**Mn-BTT**	2078.4
**Cr-BTT**	1930.4
**Cu-BTT**	1755.9

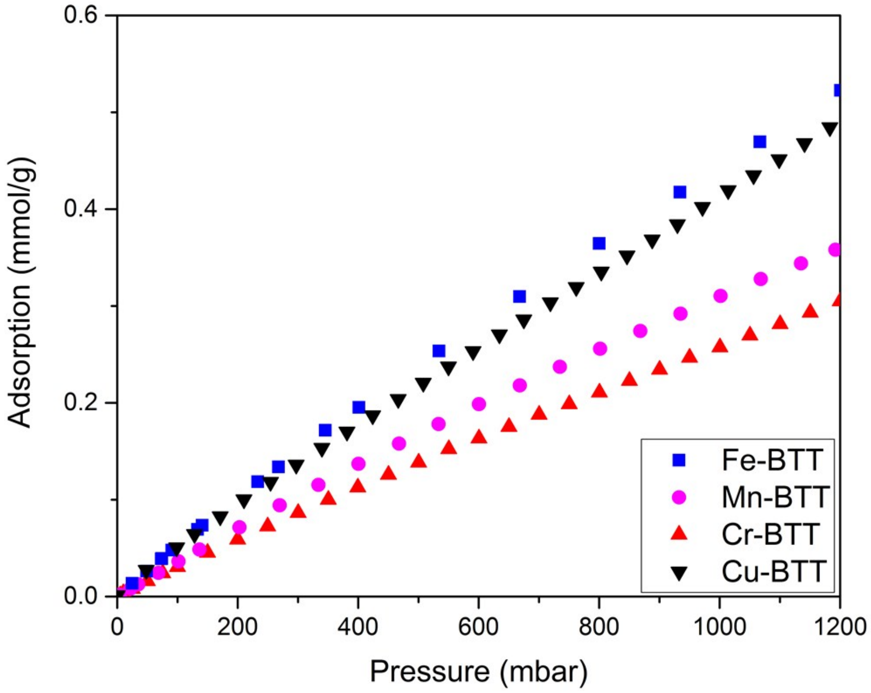

Figure S2. Nitrogen adsorption of M-BTT analogues at 298 K. A comparison of the N_2 uptake with CO_2 uptake at the same temperature (Figures S3-S6) reveals selective adsorption of CO_2 over N_2, which is favorable in the context of post-combustion CO_2 capture.

Fitting M-BTT CO_2 adsorption isotherms

The CO_2 adsorption isotherms collected at 298, 308, and 318 K were fitted with a dual-site Langmuir model (Eq. S1):

$$n = \frac{q_{sat,1} b_1 P}{1 + b_1 P} + \frac{q_{sat,2} b_2 P}{1 + b_2 P} \qquad \text{(S1)}$$

where n is adsorbed CO_2 in mmol/g, $q_{\text{sat,i}}$ is the adsorption capacity for the site i, b_{i} is the Langmuir parameter for this site, and P is the pressure in bar. The fitted parameters for each adsorption isotherm can be found in Tables S2-S5, and a comparison of the experimental CO_2 adsorption isotherms with the fitted dual-site Langmuir model based on the experimental data

is shown in Fig. S3-S6. The dual-site Langmuir model was chosen because of the validity of the theory behind this model for prediction of the interaction of heterogeneous surfaces with gas-phase small molecules. For porous materials that have adsorption sites of differing binding energies, a single-site Langmuir model is unable to accurately predict the adsorption behavior. The use of a dual-site Langmuir model for fitting gas adsorption isotherm data and the extraction of isosteric heats of adsorption has been reported elsewhere.[2-4]

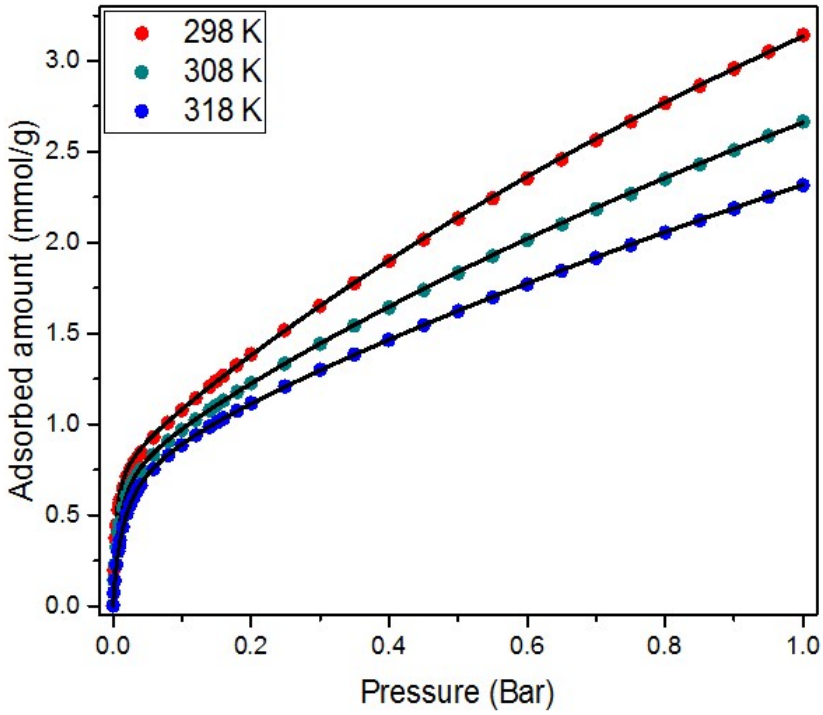

Figure S3. Dual-site Langmuir fits for CO_2 adsorption in Fe-BTT at 298, 308, and 318 K.

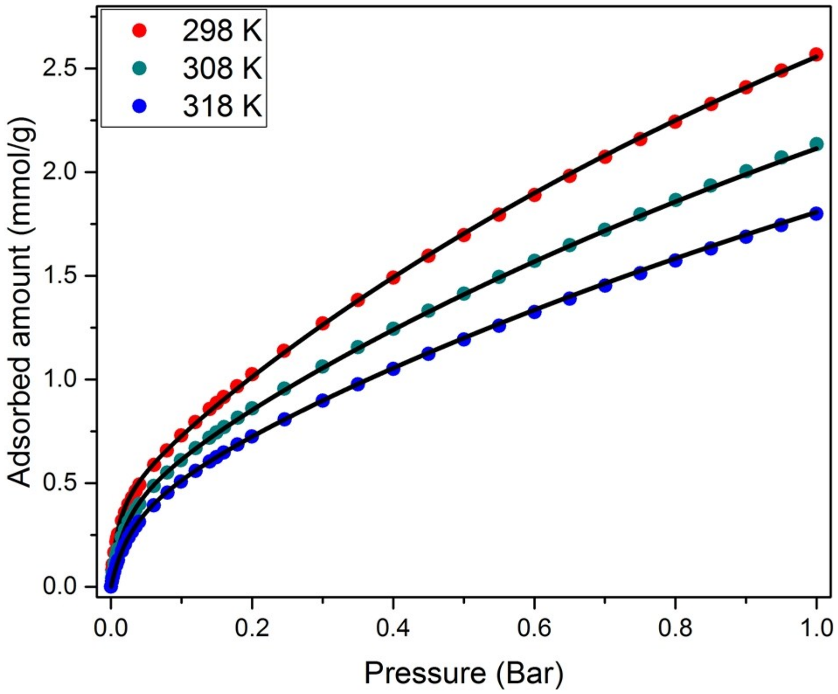

Figure S4. Dual-site Langmuir fits for CO_2 adsorption in Mn-BTT at 298, 308, and 318 K.

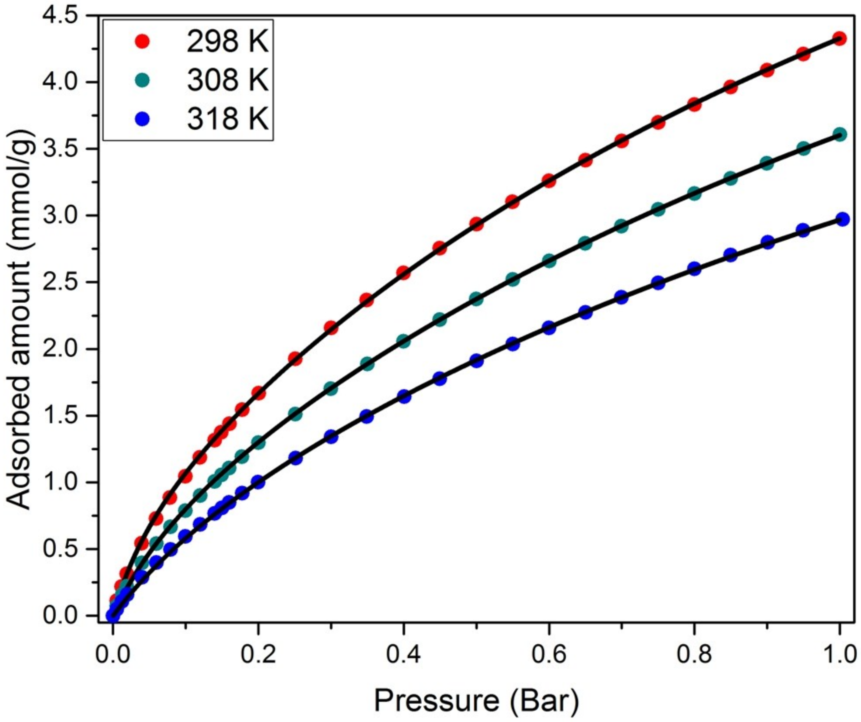

Figure S5. Dual-site Langmuir fits for CO_2 adsorption in Cr-BTT at 298, 308, and 318 K.

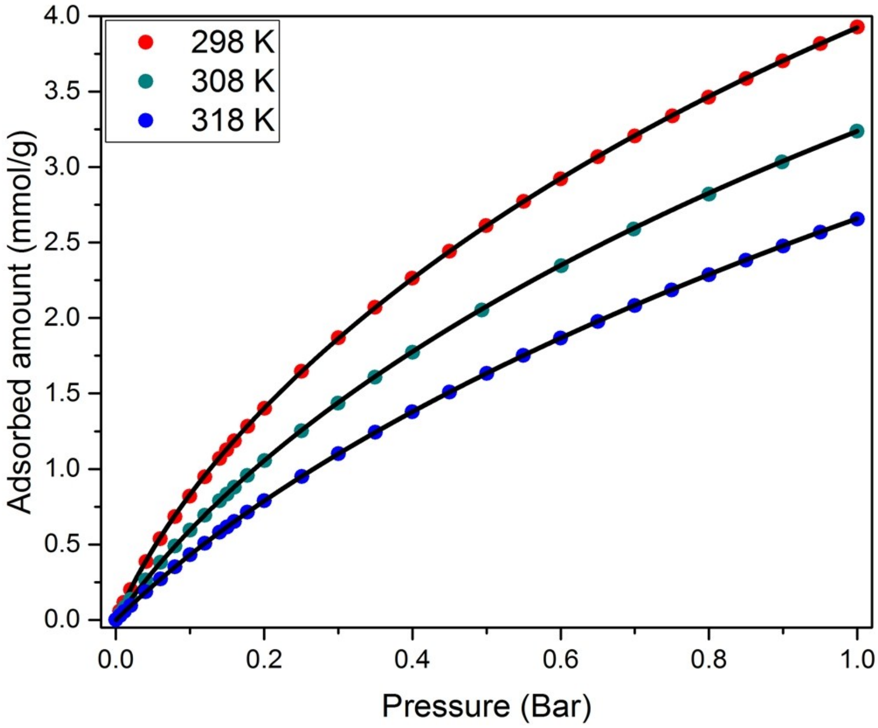

Figure S6. Dual-site Langmuir fits for CO_2 adsorption in Cu-BTT at 298, 308, and 318 K.

Table S2. Dual-site Langmuir parameters for CO_2 adsorption in M-BTT.

Framework	Temperature (K)	q_{sat1}	b_1	q_{sat2}	b_2
Fe-BTT	298	0.0008056	2.82	0.009	0.0035
	308	0.0008	1.232047	0.008278	0.002914
	318	0.000813	0.685	0.008293	0.002237
Mn-BTT	298	0.00048	1.051007	0.006683	0.004522
	308	0.0008	1.232047	0.008278	0.002914
	318	0.000813	0.685	0.008293	0.002237
Cr-BTT	298	0.00087	0.166593	0.009462	0.00589
	308	0.000773	0.102999	0.00899	0.004756
	318	0.000715	0.0565	0.00785	0.0043
Cu-BTT	298	0.000866	0.071123	0.008669	0.005748
	308	0.000877	0.038788	0.00832	0.004395
	318	0.000723	0.028083	0.00868	0.003243

Calculating Isosteric heats of adsorption

The Clausius-Clapeyron equation (Eqn. S2) was used to calculate the isosteric heats of adsorption, $-Q_{st}$, for CO_2 adsorption in M-BTT analogs at 298, 308 K, and 318 K, using dual-site Langmuir-Freundlich fits.

$$(ln\ P)_n = -\frac{Q_{st}}{R}\left(\frac{1}{T}\right) + C \qquad \text{(S2)}$$

Where, P is the pressure in any desired unit, n is the amount adsorbed mol/g, T is the temperature in K, R is the universal gas constant kJ/mol·K, and C is a constant. The isosteric heat of adsorption, $-Q_{st}$, was obtained from the slope of plots of $(\ln P)_n$ as a function of inverse T. The isosteric heats of adsorption as a function of CO_2 loading in M-BTT can be found in Figure 2 in the main text.

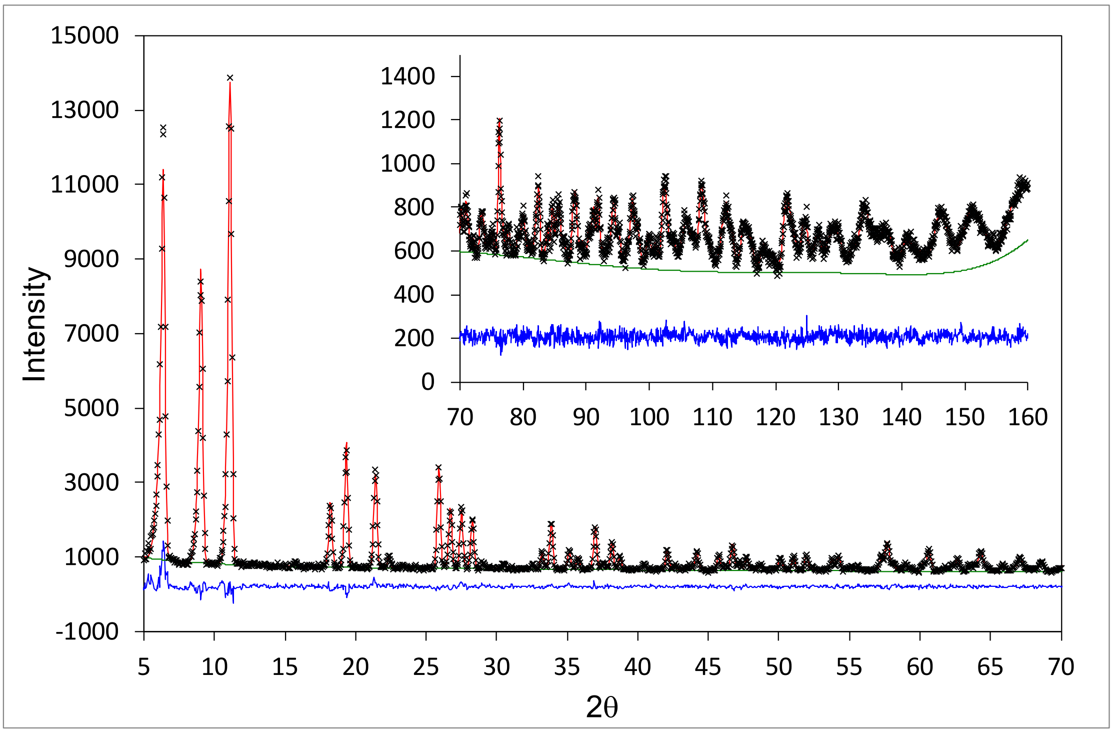

Figure S7. Neutron powder diffraction data collected on an activated sample of Cu-BTT at 10 K. The green line, crosses, and red line represent the background, experimental, and calculated diffraction patterns, respectively. The blue line represents the difference between experimental and calculated patterns.

Table S3. Fractional atomic coordinates, occupancies, and isotropic displacement parameters obtained from Rietveld refinement of the activated Cu-BTT structural model against the data presented in Figure S7. The optimal refinement yielded a cubic structure with Pm-3m space group, a = 18.5198(4) Å, and V = 6351.9(4) Å^3. Values in parentheses indicate standard deviation. Goodness-of-fit (GOF) parameters: χ^2= 0.99, wRp = 0.0354, Rp = 0.0308. (The number for corresponding CIF file in CCDC database: 1582009).

Atom	x	Y	Z	occupancy	Uiso*100 (Å^2)
Cu1	0.1322(5)	0.5	0.0	1.0	0.010(3)
C1	0.1483(3)	0.3371(3)	−0.1483(3)	1.0	0.0063(8)
C2	0.1808(2)	0.2735(3)	−0.1808(2)	1.0	0.0063(8)
C3	0.2416(3)	0.2416(3)	−0.1492(4)	1.0	0.0063(8)
Cl1	0.0	0.5	0.0	1.0	0.045(5)
N1	0.1296(2)	0.4251(2)	−0.0787(2)	1.0	0.0102(5)
N2	0.1747(2)	0.3704(2)	−0.0892(2)	1.0	0.0102(5)
H1	0.2664(5)	0.2664(5)	−0.1027(7)	1.0	0.021(4)
Cu2	0.231(2)	0.356(3)	0.0	0.192(8)	0.06409
Os	0.250(3)	0.5	0.0	0.17(2)	0.01168
Cl2	0.366(2)	0.366(2)	0.0	0.192(8)	0.06535

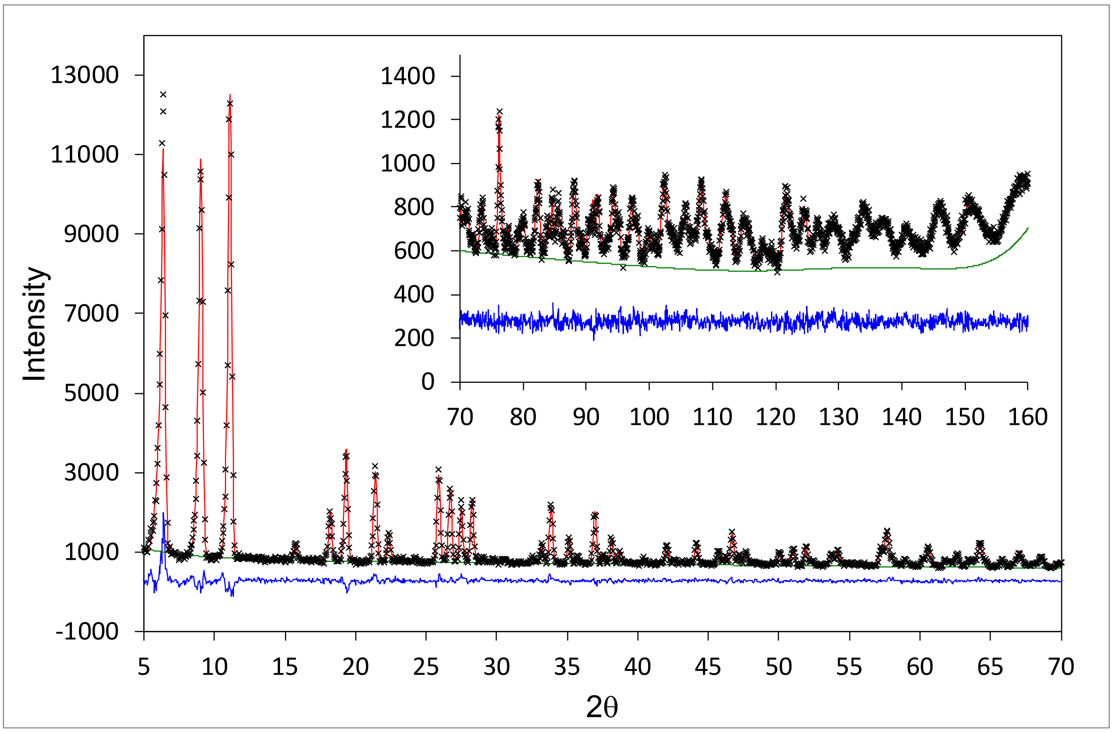

Figure S8. Neutron powder diffraction data collected at 10 K for a sample of Cu-BTT loaded with 0.533 CO_2 per Cu site (6.396 CO_2 per unit cell). The green line, crosses, and red line represent the background, experimental, and calculated diffraction patterns, respectively. The blue line represents the difference between experimental and calculated patterns.

Table S4. Fractional atomic coordinates, occupancies, and isotropic displacement parameters obtained from Rietveld refinement of the structural model for Cu-BTT (loaded with 0.533 CO_2 per Cu site) against diffraction data presented in Figure S8. The structure is cubic with Pm-3m space group, a = 18.5295(3) Å, and V = 6362.0(3) Å^3. Values in parentheses represent standard deviation. GOF parameters: χ^2= 1.01, wRp = 0.0354, Rp = 0.0305. (The number for corresponding CIF file in CCDC database: 1582006).

Atom	x	y	Z	occupancy	Uiso*100 (Å^2)
Cu1	0.1330(5)	0.5	0	1	0.008(3)
C1	0.1477(3)	0.3374(3)	−0.1477(3)	1	0.0065(9)
C2	0.1801(2)	0.2714(3)	−0.1801(2)	1	0.0065(9)
C3	0.2420(3)	0.2420(3)	−0.1484(4)	1	0.0065(9)
Cl1	0	0.5	0	1	0.041(5)
N1	0.1294(2)	0.4254(2)	−0.0784(2)	1	0.0081(5)
N2	0.1741(2)	0.3701(2)	−0.0894(2)	1	0.0081(5)
H1	0.2657(5)	0.2657(5)	−0.1013(7)	1	0.020(4)
Cu2	0.227(1)	0.384(2)	0	0.19199	0.06409
C1a	0.291(1)	0.4388(6)	0	0.266(5)	0.011(6)
O1a	0.274(2)	0.5	0	0.533(9)	0.08(1)
O1b	0.312(2)	0.380(1)	0	0.266(5)	0.05(1)
Cl2	0.358(2)	0.358(2)	0	0.19199	0.06535
Os	0.2505(7)	0.5	0	0.17378	0.01168

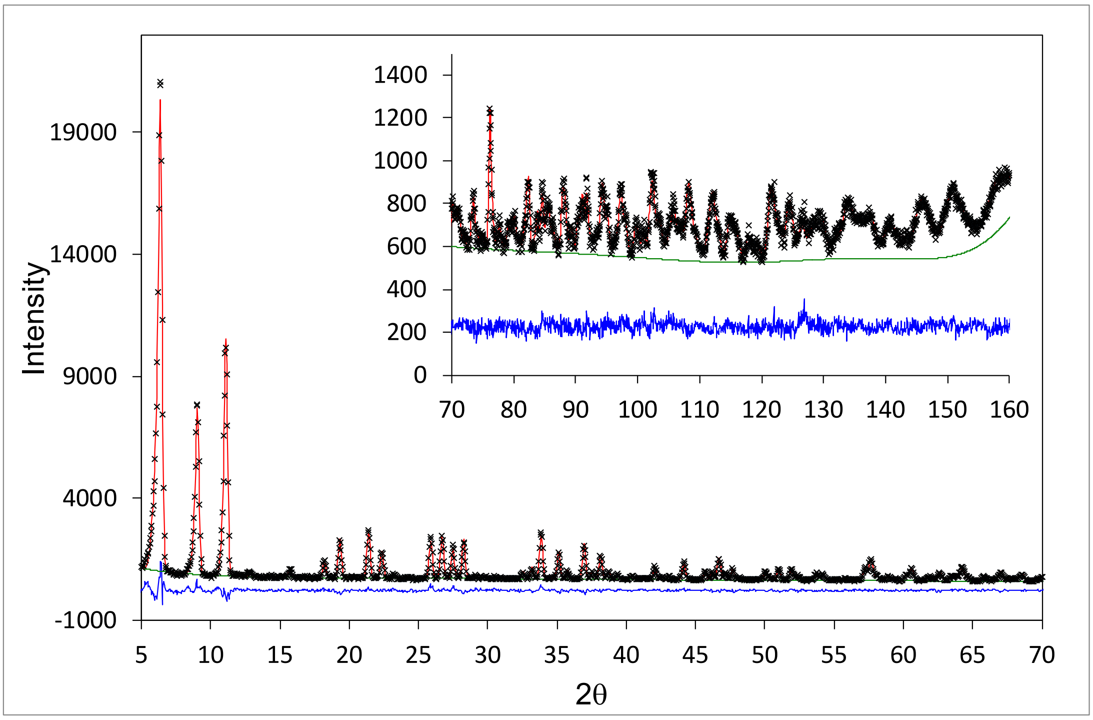

Figure S9. Neutron powder diffraction data collected at 10 K on a sample of Cu-BTT loaded with 1.558 CO2 per Cu site (18.696 CO_2 per unit cell). The green line, crosses, and red line represent the background, experimental, and calculated diffraction patterns, respectively. The blue line represents the difference between experimental and calculated patterns.

Table S5. Fractional atomic coordinates, occupancies, and isotropic displacement parameters obtained from Rietveld refinement of the structural model for Cu-BTT loaded with 1.558 CO2 per Cu site (18.696 CO_2 per unit cell) against diffraction data collected presented in Figure S9. The structure is cubic, space group Pm-3m, a = 18.5323(4) Å,V = 6364.8(4) Å^3. Values in parentheses represent standard deviation. GOF parameters: χ^2= 1.05, wRp = 0.0369, Rp = 0.0319. (The number for corresponding CIF file in CCDC database: 1582007).

Atom	x	y	Z	occupancy	Uiso*100 (Å^2)
Cu1	0.1330(5)	0.5	0	1	0.016(3)
C1	0.1478(3)	0.3363(3)	−0.1478(3)	1	0.0085(8)
C2	0.1793(3)	0.2717(4)	−0.1793(3)	1	0.0085(8)
C3	0.2405(3)	0.2405(3)	−0.1474(4)	1	0.0085(8)
Cl1	0	0.5	0	1	0.061(6)
N1	0.1299(2)	0.4258(2)	−0.0778(2)	1	0.0103(5)
N2	0.1744(2)	0.3699(2)	−0.0890(2)	1	0.0103(5)
H1	0.2636(5)	0.2636(5)	−0.1014(7)	1	0.016(3)
Cu2	0.216(1)	0.328(2)	0	0.19199	0.06409
C1a	0.2915(7)	0.4389(4)	0	0.434(5)	0.03625
O1a	0.2753(8)	0.5	0	0.87(1)	0.10109
O1b	0.309(1)	0.3787(7)	0	0.434(5)	0.07825
C4a	0.095(4)	0.066(3)	0.066(3)	0.058(3)	0.04533
O4a	0.080(5)	0.023(3)	0.023(3)	0.058(3)	0.04533
O4b	0.110(3)	0.110(3)	0.110(3)	0.17(1)	0.04533
C2a	0	0.272(1)	0.036(1)	0.180(4)	0.03761
O2b	0	0.221(1)	0.072(2)	0.180(4)	0.06551
O2a	0	0.324(1)	0	0.72(2)	0.05482
C3a	0.1597(8)	0.1597(8)	0	0.33(1)	0.06602
O3a	0.0990(8)	0.173(4)	0	0.165(5)	0.04786
O3b	0.2199(8)	0.146(4)	0	0.165(5)	0.04786
Os	0.2505(8)	0.5	0	0.17378	0.01168
Cl2	0.349(2)	0.349(2)	0	0.19199	0.06535

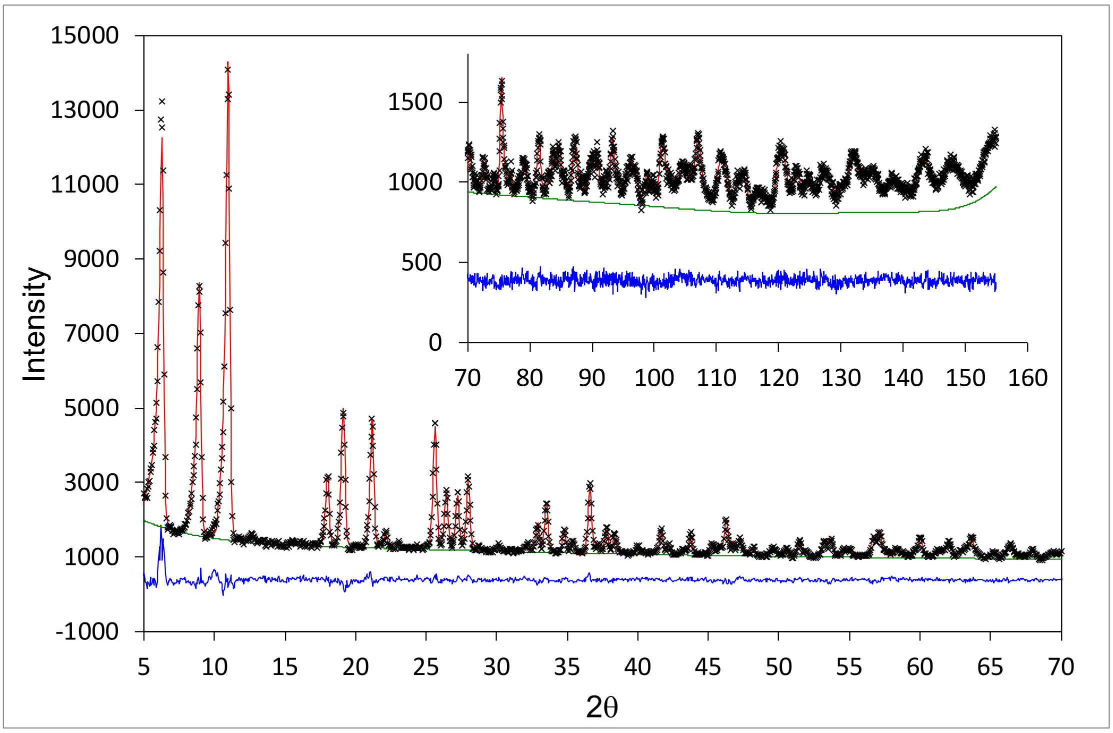

Figure S10. Neutron powder diffraction data collected at 10 K for an activated sample of Cr-BTT. The green line, crosses, and red line represent the background, experimental, and calculated diffraction patterns, respectively. The blue line represents the difference between experimental and calculated patterns.

Table S6. Fractional atomic coordinates, occupancies, and isotropic displacement parameters obtained from Rietveld refinement of the activated Cr-BTT structural model against data presented in Figure S10. The structure is cubic, space group Pm-3m, a = 18.6689(4) Å, and V = 6506.6(4) Å^3. Values in parentheses indicate one standard deviation in the refined value. GOF parameters: χ^2 = 1.06, wRp = 0.0296, Rp = 0.0255. (The number for corresponding CIF file in CCDC database: 1582001).

Atom	X	y	Z	occupancy	Uiso*100 (Å^2)
Cr1	0.136(1)	0.5	0	1	0.00605
C1	0.1494(3)	0.3365(4)	0.1494(3)	1	0.009(1)
C2	0.1817(3)	0.2737(4)	0.1817(3)	1	0.009(1)
C3	0.2435(3)	0.2435(3)	−0.1507(4)	1	0.009(1)
Cl1	0	0.5	0	1	0.032(5)
N1	0.1311(2)	0.4234(2)	0.0801(2)	1	0.0119(6)
N2	0.1750(2)	0.3694(2)	0.0909(2)	1	0.0119(6)
H1	0.2675(5)	0.2675(5)	−0.1062(7)	1	0.016(4)
Os	0.233(2)	0.5	0	0.24(2)	0.00351

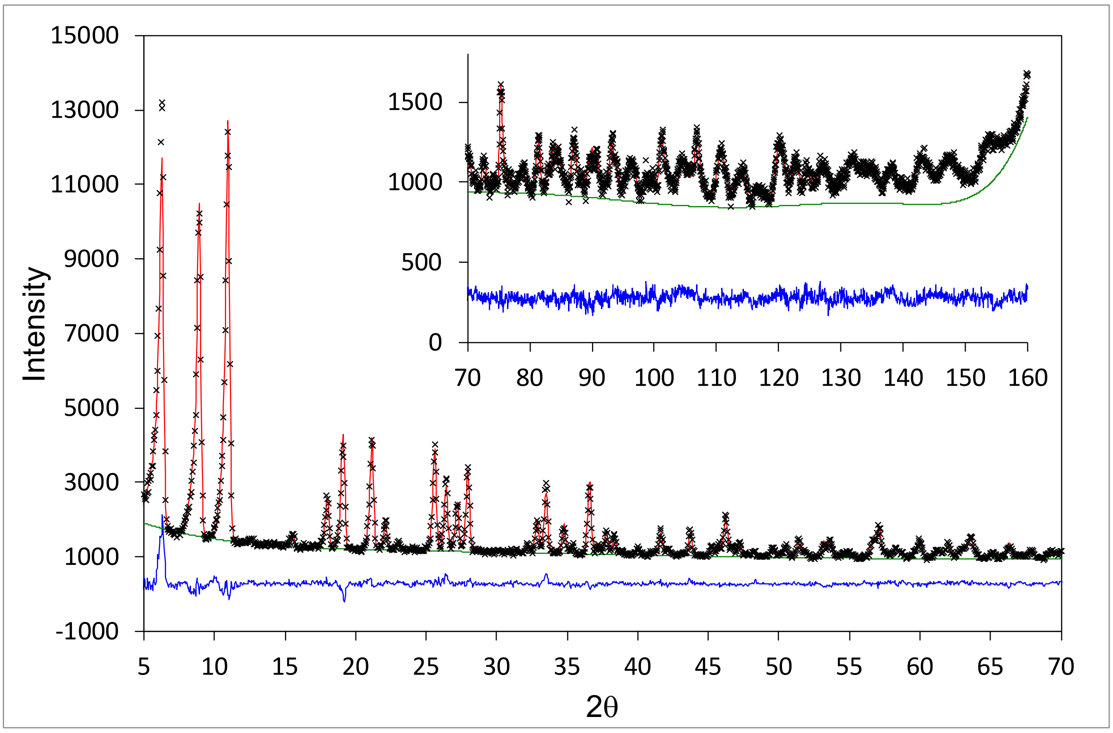

Figure S11. Neutron powder diffraction data collected at 10 K on a sample of Cr-BTT loaded with 0.56 CO_2 per Cr site (6.72 CO_2 per unit cell). The green line, crosses, and red line represent the background, experimental, and calculated diffraction patterns, respectively. The blue line represents the difference between experimental and calculated patterns. Data was collected at NIST.

Table S7. Fractional atomic coordinates, occupancies, and isotropic displacement parameters obtained from Rietveld refinement of the structural model for Cr-BTT loaded with 0.56 CO_2 per Cr site (6.72 CO_2 per unit cell) against the data presented in Figure S11. The structure is cubic, space group Pm-3m, a = 18.6867(5) Å, V = 6252.2(5) Å^3. Values in parentheses indicate standard deviation. GOF parameters: χ^2 = 1.16, wRp = 0.0333, Rp = 0.0288. (The number for corresponding CIF file in CCDC database: 1582003).

Atom	X	Y	Z	occupancy	Uiso*100 (Å^2)
Cr1	0.132(1)	0.5	0	1	0.00991
C1	0.1485(3)	0.3366(4)	-0.1485(3)	1	0.013(1)
C2	0.1806(3)	0.2713(4)	-0.1806(3)	1	0.013(1)
C3	0.2425(3)	0.2425(3)	-0.1486(5)	1	0.013(1)
Cl1	0	0.5	0	1	0.057(7)
N1	0.1301(2)	0.4231(2)	-0.0792(2)	1	0.0139(7)
N2	0.1753(3)	0.3698(2)	-0.0900(2)	1	0.0139(7)
H1	0.2662(6)	0.2662(6)	-0.1068(8)	1	0.016(5)
Os	0.230(1)	0.5	0	0.2353	0.00351
Ca	0.291(1)	0.4399(7)	0	0.277(5)	0.07288
Oa	0.275(2)	0.5	0	0.56(1)	0.08225
Ob	0.312(2)	0.382(1)	0	0.277(5)	0.07411

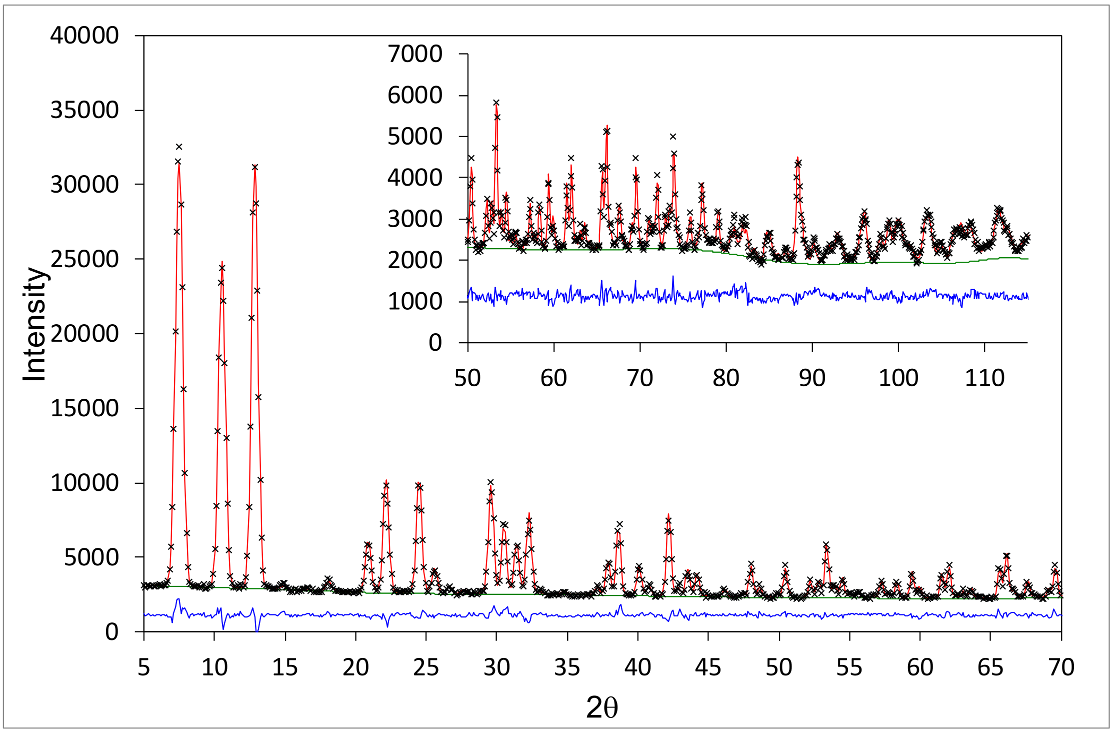

Figure S12. Neutron powder diffraction data collected at 10 K on Cr-BTT loaded with 0.436 CO_2 per Cr site (5.23 CO_2 per unit cell). The green line, crosses, and red line represent the background, experimental, and calculated diffraction patterns, respectively. The blue line represents the difference between experimental and calculated patterns. Data was collected at Chalk River Laboratories at the Canadian Neutron Beam Center.

Table S8. Fractional atomic coordinates, occupancies, and isotropic displacement parameters obtained from Rietveld refinement of the structural model for Cr-BTT loaded with 0.436 CO_2 per Cr site (5.23 CO_2 per unit cell) against the data presented in Figure S12. The structure is cubic, space group Pm-3m, a = 18.7761(6) Å, V = 6619.4(6) Å^3. Values in parentheses indicate standard deviation. GOF parameters: χ^2 = 2.13, wRp = 0.0360, Rp = 0.0284. (The number for corresponding CIF file in CCDC database: 1582004).

Atom	X	Y	Z	occ.	Uiso*100 (Å^2)
Cr1	0.134(2)	0.5	0	1	0.01(3)
C1	0.1476(5)	0.333(1)	−0.1476(5)	1	0.012(3)
C2	0.1811(5)	0.2703(6)	−0.1811(5)	1	0.012(3)
C3	0.2440(5)	0.2440(5)	−0.1484(6)	1	0.012(3)
Cl1	0	0.5	0	1	0.12(2)
N1	0.1314(3)	0.4221(3)	−0.0788(3)	1	0.010(1)
N2	0.1748(4)	0.3695(3)	−0.0924(4)	1	0.010(1)
H1	0.2635(7)	0.2635(7)	−0.1027(9)	1	0.02(1)
Os	0.233(2)	0.5	0	0.22	0.004
Ca	0.289(2)	0.4385(8)	0	0.218(5)	0.012(3)
Oa	0.277(2)	0.5	0	0.44(1)	0.018(2)
Ob	0.306(3)	0.378(1)	0	0.218(5)	0.012(2)

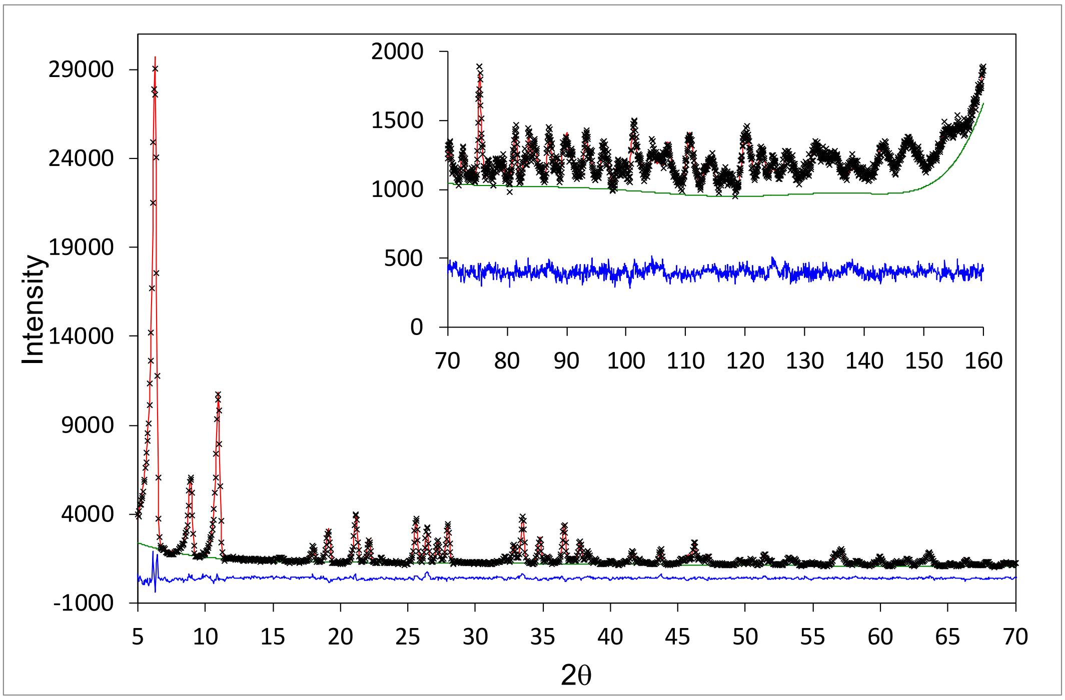

Figure S13. Neutron powder diffraction data collected at 10 K on Cr-BTT loaded with 1.275 CO_2 per Cr site (15.304 CO_2 per unit cell). The green line, crosses, and red line represent the background, experimental, and calculated diffraction patterns, respectively. The blue line represents the difference between experimental and calculated patterns.

Table S9. Fractional atomic coordinates, occupancies, and isotropic displacement parameters obtained from Rietveld refinement of the structural model for Cr-BTT loaded with 1.275 CO_2 per Cr site (15.304 CO_2 per unit cell) against data shown in Figure S13. The structure is cubic, space grupd Pm-3m, a =18.6816(3) Å, V = 6520.0(4) Å^3. Values in parentheses indicate standard deviation. GOF parameters: χ^2 = 1.18, wRp = 0.0317, Rp = 0.0271. (The number for corresponding CIF file in CCDC database: 1582002).

Atom	x	y	Z	occupancy	Uiso*100 (Å^2)
Cr1	0.131(1)	0.5	0	1	0.00372
C1	0.1488(3)	0.3368(4)	−0.1488(3)	1	0.0111(9)
C2	0.1779(3)	0.2690(4)	−0.1778(3)	1	0.0111(9)
C3	0.2410(3)	0.2410(3)	−0.1460(5)	1	0.0111(9)
Cl1	0	0.5	0	1	0.11(1)
N1	0.1306(2)	0.4223(2)	−0.0788(2)	1	0.012(5)
N2	0.1748(2)	0.3694(2)	−0.0905(2)	1	0.012(5)
H1	0.2610(6)	0.2610(6)	−0.1040(7)	1	0.010(4)
C1a	0.2907(9)	0.4397(5)	0	0.386(6)	0.10145
O1a	0.272(1)	0.5	0	0.77(1)	0.065
O1b	0.310(2)	0.3808(9)	0	0.386(6)	0.10486
C4a	0.114(4)	0.0641(9)	0.0641(9)	0.133(5)	0.10621
O4a	0.071(4)	0.034(3)	0.034(3)	0.133(5)	0.10621
O4b	0.158(4)	0.095(3)	0.095(3)	0.133(5)	0.10621
C2a	0	0.277(2)	0.031(1)	0.187(4)	0.03831
O2b	0	0.224(2)	0.062(2)	0.187(4)	0.05208
O2a	0	0.330(2)	0	0.75(2)	0.06768
C3a	0.1656(7)	0.1656(7)	0	0.50(1)	0.0634
O3a	0.111(1)	0.193(2)	0	0.250(6)	0.0634
O3b	0.220(1)	0.137(2)	0	0.250(6)	0.0634
Os	0.229(1)	0.5	0	0.2353	0.00351

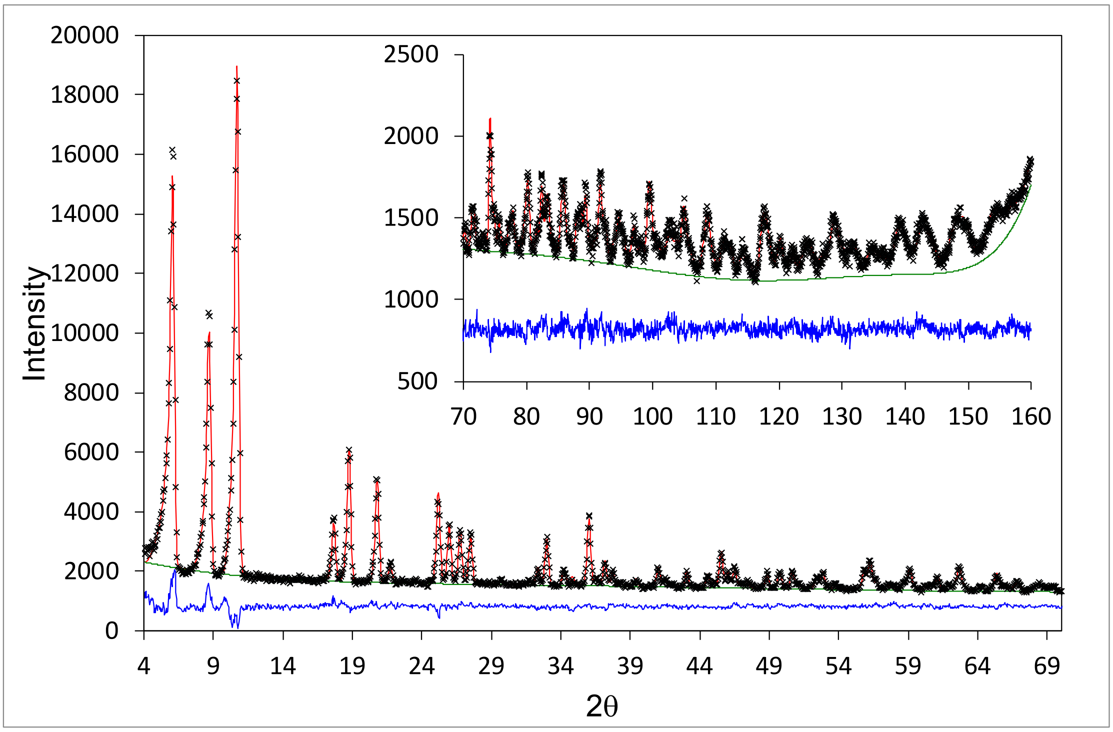

Figure S14. Neutron powder diffraction data obtained at 10 K on an activated sample of Fe-BTT. The green line, crosses, and red line represent the background, experimental, and calculated diffraction patterns, respectively. The blue line represents the difference between experimental and calculated patterns.

Table S10. Fractional atomic coordinates, occupancies, and isotropic displacement parameters obtained from Rietveld refinement of the activated Fe-BTT structural model against the data presented in Figure S14. The structure is cubic, space group Pm-3m, a =18.8957(4) Å, V = 6746.6(5) Å^3. Values in parentheses indicate standard deviation. GOF parameters: χ^2 = 1.3, wRp = 0.0272, Rp = 0.0236. (The number for corresponding CIF file in CCDC database: 1582010).

Atom	X	y	Z	occupancy	Uiso*100 (Å^2)
Fe1	0.1343(4)	0.5	0	1	0.006(2)
C1	0.1512(3)	0.3361(5)	−0.1512(3)	1	0.019(1)
C2	0.1807(3)	0.2710(4)	−0.1807(3)	1	0.019(1)
C3	0.2419(3)	0.2419(3)	−0.1466(4)	1	0.019(1)
Cl1	0	0.5	0	1	0.007(3)
N1	0.1303(2)	0.4208(2)	−0.0816(2)	1	0.0161(7)
N2	0.1758(2)	0.3682(2)	−0.0913(2)	1	0.0161(7)
H1	0.2629(5)	0.2629(5)	−0.1069(6)	1	0.028(5)
Fe2	0.264(5)	0	0	0.18(2)	0.10(5)
Os	0.251(1)	0.5	0	0.65(2)	0.035(6)
Cs	0.303(1)	0.5	0.055(1)	0.324(9)	0.035(6)
Hsa	0.28(2)	0.5	0.0995(9)	0.324(9)	0.11(2)
Hsb	0.330(2)	0.4578(8)	0.053(2)	0.324(9)	0.11(2)

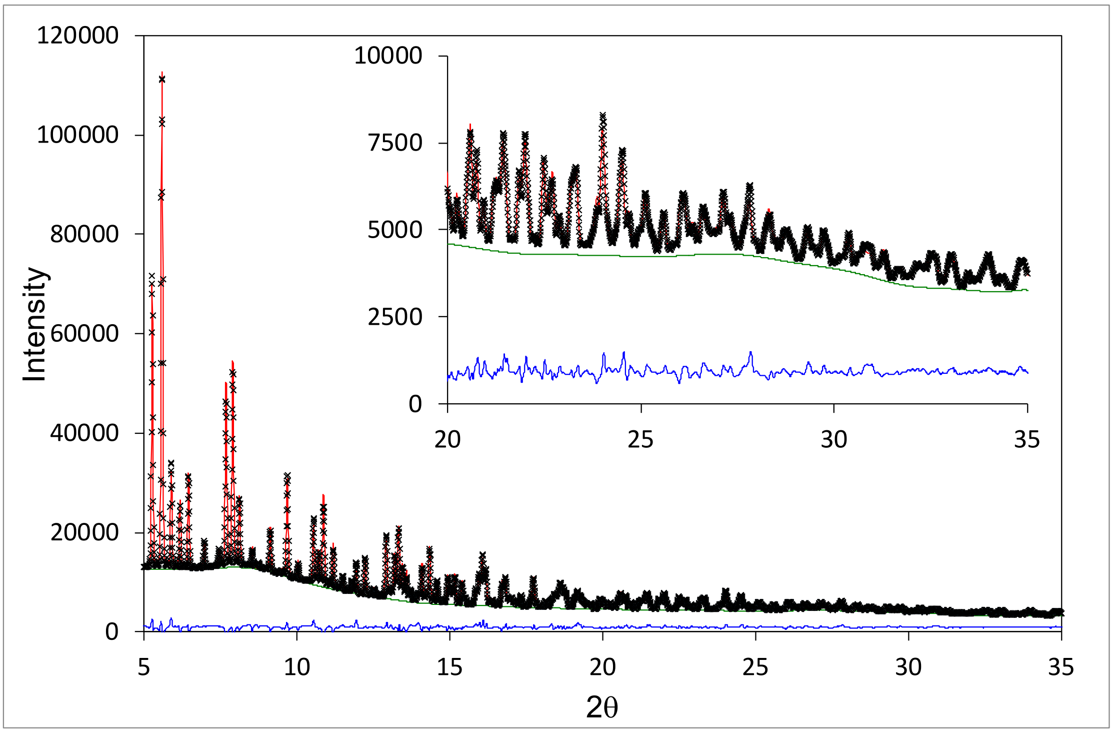

Figure 15. Synchrotron powder diffraction data obtained at 100 K on an activated sample of Fe-BTT. The green line, crosses, and red line represent the background, experimental, and calculated diffraction patterns, respectively. The blue line represents the difference between experimental and calculated patterns.

Table S11. Fractional atomic coordinates, occupancies, and isotropic displacement parameters obtained from Rietveld refinement of the activated Fe-BTT structural model against the data in Figure S15. The structure is cubic, space group Pm-3m, a = 18.8004(1) Å, V = 6645.1 (1) Å^3. Values in parentheses indicate one standard deviation in the refined value. GOF parameters: χ^2 = 3.20, wRp = 0.0289, Rp = 0.0213. (The number for corresponding CIF file in CCDC database: 1582005).

Atom	X	y	Z	occupancy	Uiso*100 (Å^2)
Fe1	0.1360(1)	0.5	0	1	0.0183(4)
C1	0.1498(4)	0.3341(5)	−0.1498(4)	1	0.048(2)
C2	0.1810(4)	0.2701(4)	−0.1810(4)	1	0.048(2)
C3	0.2397(3)	0.2397(3)	−0.1468(4)	1	0.048(2)
Cl1	0	0.5	0	1	0.015(2)
N1	0.1294(1)	0.4201(2)	−0.0795(2)	1	0.019(1)
N2	0.1746(2)	0.3661(2)	−0.0920(2)	1	0.019(1)
Fe3	0.2447(24)	0	0	0.114(2)	0.28059
Os	0.2578(5)	0.5	0	1	0.09725
Cs	0.3180(6)	0.5	0.0491(7)	0.5	0.09725
Os2	0.370(3)	0.370(3)	0	0.0636(7)	0.12388
Cs2	0.4194(22)	0.4194(22)	−0.031(8)	0.0636(7)	0.12388
Os3	−0.086(5)	0	0	0.114(2)	0.28059
Fe2	0.2523(8)	0.3619(7)	0	0.127(1)	0.12388

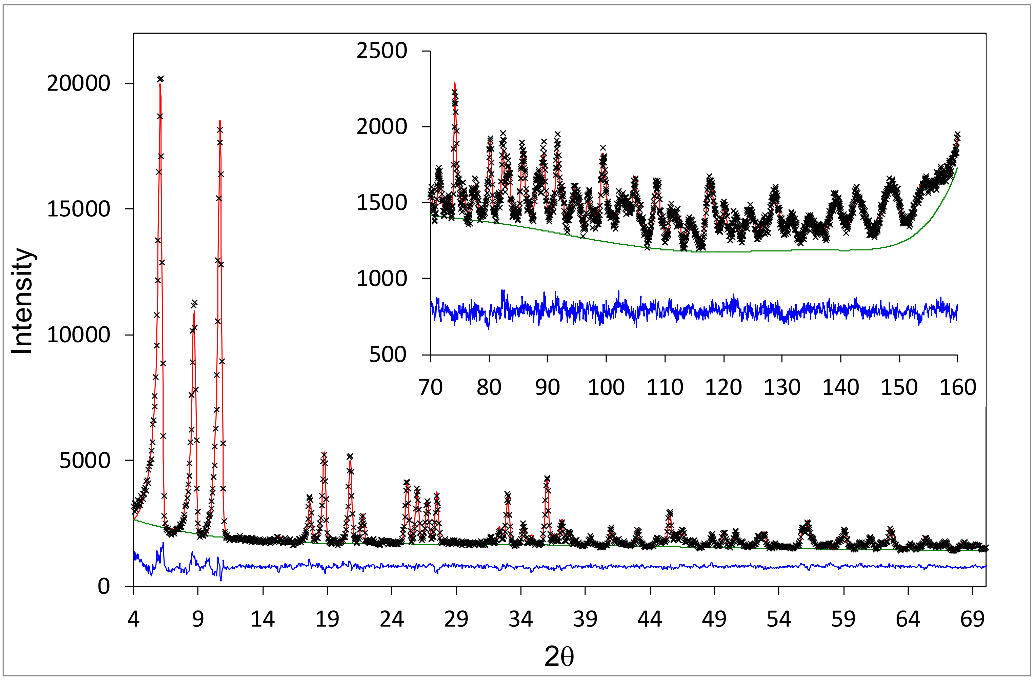

Figure S16. Neutron powder diffraction data obtained at 10 K on a sample of Fe-BTT loaded with 0.662 CO_2 per Fe site (7.944 CO_2 per unit cell). The green line, crosses, and red line represent the background, experimental, and calculated diffraction patterns, respectively. The difference between experimental and calculated patterns is represented as a blue line.

Table S12. Fractional atomic coordinates, occupancies, and isotropic displacement parameters obtained from Rietveld refinement of the structural model for Fe-BTT loaded with 0.662 CO_2 per Fe site (7.944 CO_2 per unit cell) against the data presented in Figure S16. The structure is cubic, space group Pm-3m, a = 18.9005(3) Å, V = 6751.8(3) Å^3. Values in parentheses indicate standard deviation. GOF parameters: χ^2 = 1.12, wRp = 0.0272, Rp = 0.0234. (The number for corresponding CIF file in CCDC database: 1582011).

Atom	x	y	Z	occupancy	Uiso*100 (Å^2)
Fe1	0.1331(4)	0.5	0	1	0.002(1)
C1	0.1517(3)	0.3369(4)	-0.1517(3)	1	0.017(1)
C2	0.1812(3)	0.2724(4)	-0.1812(3)	1	0.017(1)
C3	0.2421(3)	0.2421(3)	-0.1470(4)	1	0.017(1)
Cl1	0	0.5	0	1	0.006(4)
N1	0.1306(2)	0.4207(2)	-0.0811(2)	1	0.0148(5)
N2	0.1766(2)	0.3680(2)	-0.0918(2)	1	0.0148(5)
H1	0.2633(5)	0.2633(5)	-0.1073(6)	1	0.025(4)
Fe2	0.268(3)	0	0	0.1793	0.10322
Os	0.2482(6)	0.5	0	0.6471	0.03508
Cs	0.301(1)	0.5	0.054(1)	0.3236	0.03508
O1a	0.258(2)	0.5	0	0.36(1)	0.09(1)
O1b	0.308(3)	0.388(2)	0	0.18(5)	0.10(2)
C1a	0.285(2)	0.445(1)	0	0.18(5)	0.10(2)
Hsa	0.277(2)	0.5	0.1009(9)	0.3236	0.10908
Hsb	0.330(1)	0.4532(8)	0.049(2)	0.3236	0.10908
C2a	0	0.285(2)	0.040(2)	0.074(4)	0.09(3)
O2b	0	0.240(5)	0.081(6)	0.074(4)	0.09(3)
O2a	0	0.3308(4)	0	0.30(2)	0.07(4)
C3a	0.135(4)	0.135(4)	0	0.152(7)	0.07(2)
O3a	0.076(5)	0.118(5)	0	0.078(4)	0.07(2)
O3b	0.194(5)	0.153(5)	0	0.078(4)	0.07(2)

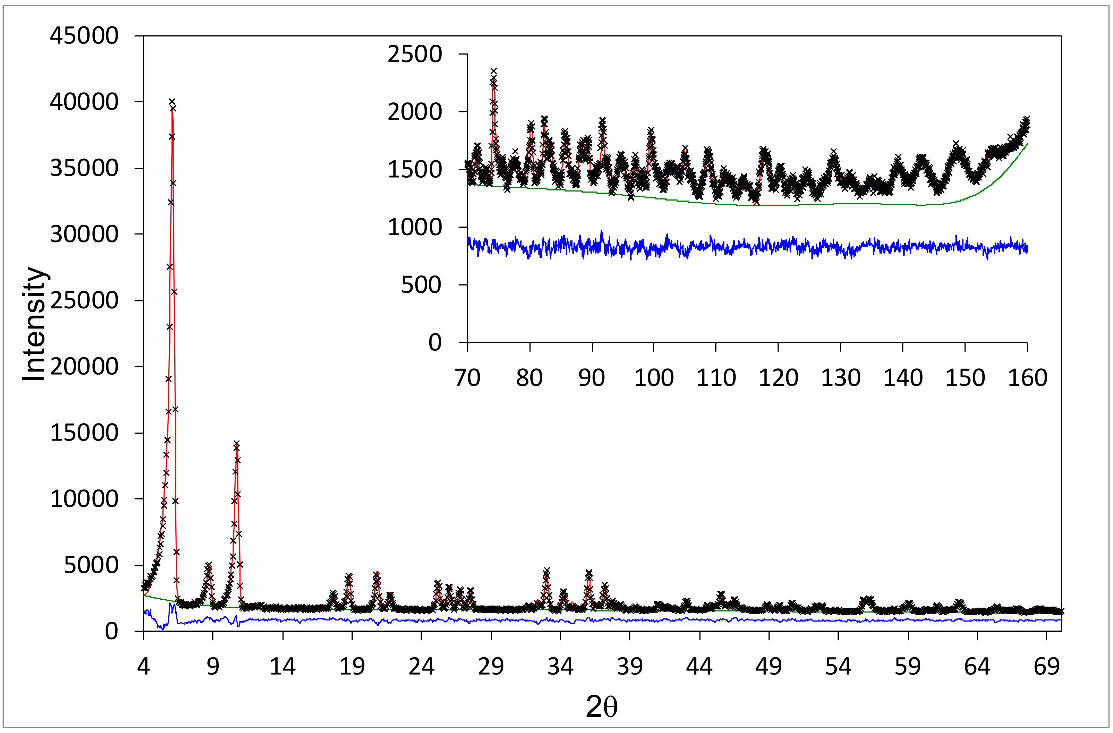

Figure S17. Neutron powder diffraction data obtained at 10 K on a sample of Fe-BTT loaded with 1.716 CO_2 per Fe site (20.592 CO_2 per unit cell). The green line, crosses, and red line represent the background, experimental, and calculated diffraction patterns, respectively. The blue line represents the difference between experimental and calculated patterns.

Table S13. Fractional atomic coordinates, occupancies, and isotropic displacement parameters obtained from Rietveld refinement of the structural model for Fe-BTT loaded with 1.716 CO_2 per Fe site (20.592 CO_2 per unit cell) against the data presented in Figure S17. The structure is cubic, space group Pm-3m, a = 18.5952(4) Å, V = 6746.1(4) Å^3. Values in parentheses indicate standard deviation. GOF parameters: χ^2 = 1.56, wRp = 0.0302, Rp = 0.0267. (The number for corresponding CIF file in CCDC database: 1582008).

Atom	X	Y	Z	occupancy	Uiso*100 (Å^2)
Fe1	0.1351(5)	0.5	0	1	0.002(2)
C1	0.1508(4)	0.3368(5)	−0.1508(4)	1	0.019(1)
C2	0.1795(4)	0.2707(5)	−0.1795(4)	1	0.019(1)
C3	0.2419(3)	0.2419(3)	−0.1473(5)	1	0.019(1)
Cl1	0	0.5	0	1	0.027(5)
N1	0.1308(3)	0.4209(3)	−0.0803(3)	1	0.0211(7)
N2	0.1752(3)	0.3665(3)	−0.0904(2)	1	0.0211(7)
H1	0.2616(6)	0.2616(6)	−0.1100(8)	1	0.026(5)
Fe2	0.2679(7)	0	0	0.1793	0.10322
Os	0.2508(7)	0.5	0	0.6471	0.03508
Cs	0.309(1)	0.5	0.047(1)	0.3236	0.03508
O1a	0.2607(8)	0.5	0	0.35765	0.09372
O1b	0.305(4)	0.385(2)	0	0.17882	0.010422
C1a	0.286(2)	0.444(1)	0	0.17882	0.010422
Hsa	0.291(2)	0.5	0.095(2)	0.3236	0.10908
Hsb	0.343(2)	0.460(2)	0.032(3)	0.3236	0.10908
C2a	0	0.277(1)	0.040(1)	0.205	0.015(6)
O2b	0	0.231(2)	0.080(2)	0.205	0.021(6)
O2a	0	0.321(1)	0	0.82	0.013(6)
C3a	0.1590(8)	0.1590(8)	0	0.67(1)	0.06(1)
O3a	0.099(1)	0.159(2)	0	0.33(1)	0.06(1)
O3b	0.218(1)	0.158(2)	0	0.33(1)	0.06(1)
C4a	0.101(2)	0.048(3)	0.048(3)	0.14(1)	0.06(2)
O4a	0.144(4)	0.079(2)	0.079(2)	0.14(1)	0.06(2)
O4b	0.055(2)	0.019(2)	0.019(2)	0.14(1)	0.06(2)

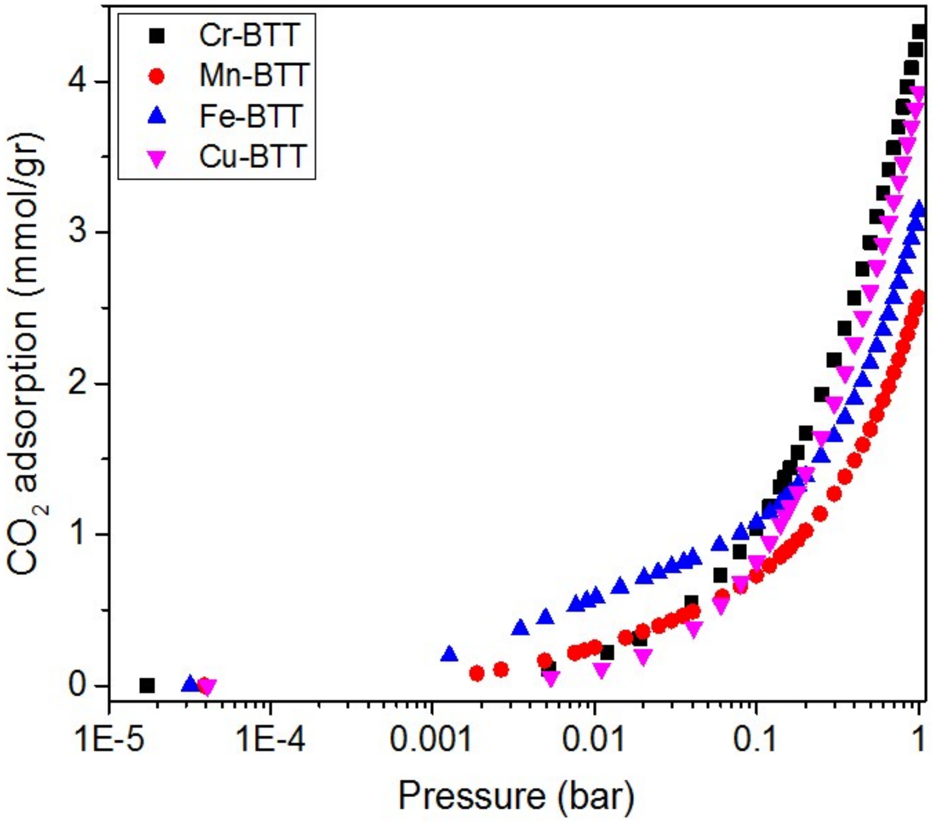

Figure S18. Semi-log scale plotted isotherm of CO_2 adsorption in M-BTT (M = Cr, Mn, Fe, and Cu).

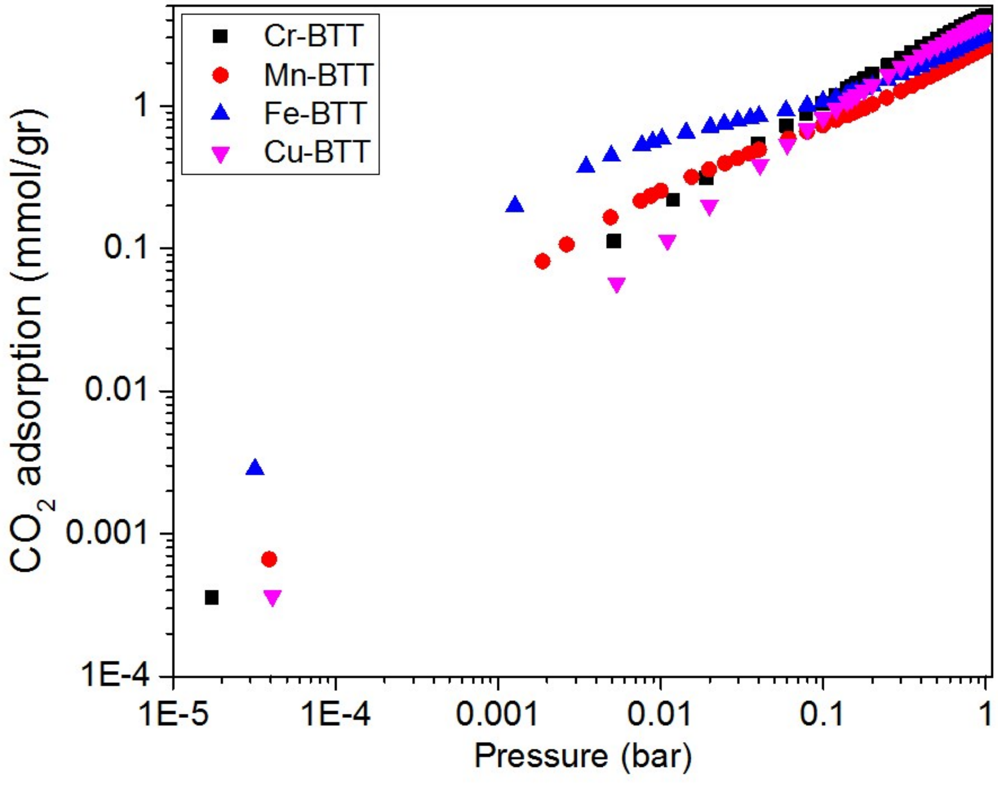

Figure S19. Log scale plotted isotherm of CO_2 adsorption in M-BTT (M= Cr, Mn, Fe, and Cu).

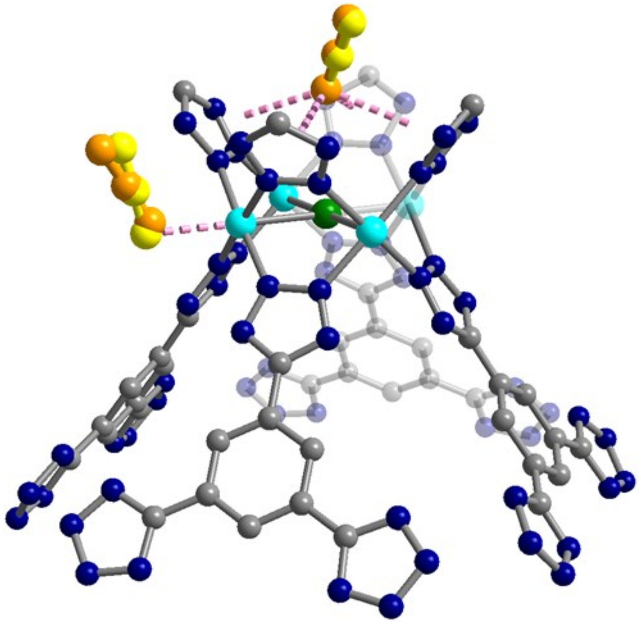

Figure S20. Locations of the primary and secondary CO_2 adsorption sites in Cu-BTT as determined from DFT calculations (orange ball and stick representation) and *in situ* neutron powder diffraction (yellow ball-and-stick representation). Cyan, green, blue, and gray spheres represent framework Cu, Cl, N, and C atoms, respectively; H atoms are omitted for clarity.

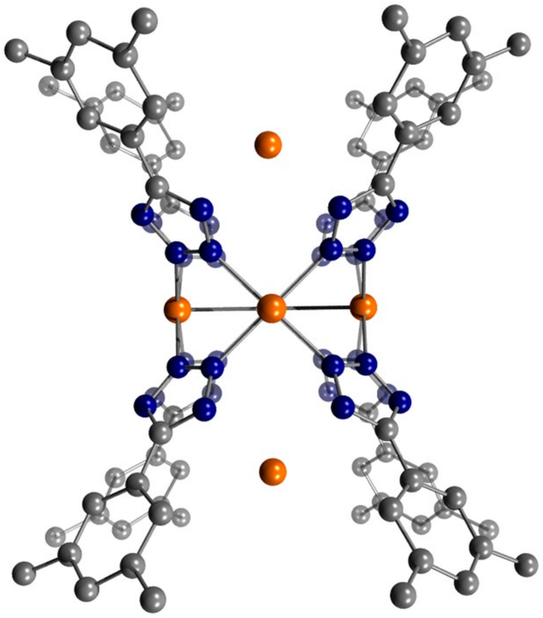

Figure S21. The position of extra-framework Fe cations inside Fe-BTT. Orange, blue, and grey spheres represent Fe, N, and C atoms, respectively; H atoms are omitted for clarity.

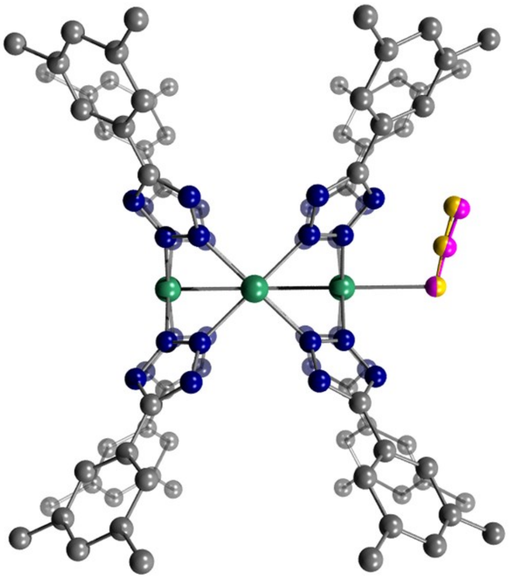

Figure S22. Comparison of the location of primary CO_2 adsorption sites in Cr-BTT as determined from analysis of diffraction data collected at NIST (pink ball-and-stick representation) and at the Chalk River Laboratories at the Canadian Neutron Beam Center (yellow ball-and-stick representation). Green, blue, and grey spheres represent Cr, N, and C atoms, respectively; H atoms are omitted for clarity.

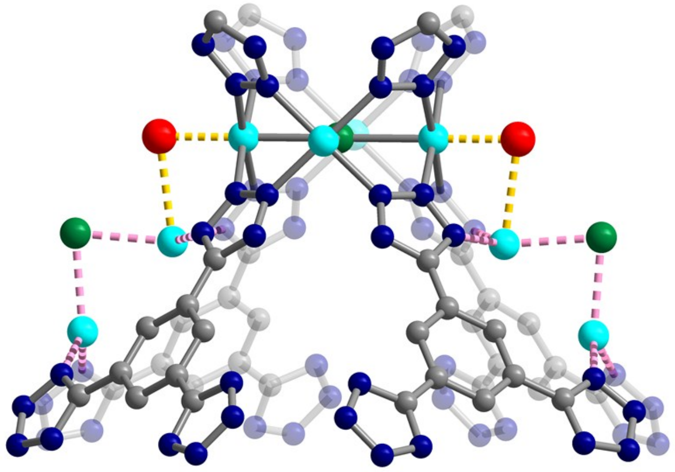

Figure S23. Extra-framework cationic clusters in Cu-BTT as determined from Rietveld analysis of high-resolution powder neutron diffraction data. Cyan, grey, blue, green and red spheres represent Cu, C, N, Cl and O atoms, respectively; H atoms are omitted for clarity. An extra-framework cationic cluster consists of two Cu and one Cl atom – waters are also observed to coordinate to neighboring Cu atoms.

References

1. S. Brunauer, P. H. Emmett and E. Teller, *Journal of the American chemical society*, 1938, **60**, 309-319.
2. J. A. Mason, K. Sumida, Z. R. Herm, R. Krishna and J. R. Long, *Energy & Environmental Science*, 2011, **4**, 3030-3040.
3. P. M. Mathias, R. Kumar, J. D. Moyer, J. M. Schork, S. R. Srinivasan, S. R. Auvil and O. Talu, *Industrial & engineering chemistry research*, 1996, **35**, 2477-2483.
4. T. M. McDonald, D. M. D'Alessandro, R. Krishna and J. R. Long, *Chemical Science*, 2011, **2**, 2022-2028.
